# Pharmacologic therapy for engraftment arrhythmia induced by transplantation of human cardiomyocytes

**DOI:** 10.1016/j.stemcr.2021.08.005

**Published:** 2021-09-09

**Authors:** Kenta Nakamura, Lauren E. Neidig, Xiulan Yang, Gerhard J. Weber, Danny El-Nachef, Hiroshi Tsuchida, Sarah Dupras, Faith A. Kalucki, Anu Jayabalu, Akiko Futakuchi-Tsuchida, Daisy S. Nakamura, Silvia Marchianò, Alessandro Bertero, Melissa R. Robinson, Kevin Cain, Dale Whittington, Rong Tian, Hans Reinecke, Lil Pabon, Björn C. Knollmann, Steven Kattman, R. Scott Thies, W. Robb MacLellan, Charles E. Murry

**Affiliations:** 1 Institute for Stem Cell and Regenerative Medicine, University of Washington, 850 Republican Street, Brotman Building Room 453, Seattle, WA 98109, USA; 2 Center for Cardiovascular Biology, University of Washington, Seattle, WA 98109, USA; 3 Division of Cardiology, Department of Medicine, University of Washington, Seattle, WA 98195, USA; 4 Department of Comparative Medicine, University of Washington, Seattle, WA 98195, USA; 5 Department of Laboratory Medicine & Pathology, University of Washington, Seattle, WA 98195, USA; 6 Sana Biotechnology, Seattle, WA 98102, USA; 7 Department of Biostatics, University of Washington, Seattle, WA 98195, USA; 8 Department of Medicinal Chemistry, University of Washington, Seattle, WA 98195, USA; 9 Department of Anesthesiology & Pain Medicine, University of Washington, Seattle, WA 98195, USA; 10 Department of Bioengineering, University of Washington, Seattle, WA 98195, USA; 11 Division of Clinical Pharmacology, Vanderbilt University School of Medicine, Nashville, TN 37232, USA

**Keywords:** cardiac cell therapy, embryonic stem cells, engraftment arrhythmia, cardiac regeneration, myocardial infarction, electrophysiology, heart failure, cardiac remuscularization, sudden cardiac death, antiarrhythmic drugs

## Abstract

Heart failure remains a significant cause of morbidity and mortality following myocardial infarction. Cardiac remuscularization with transplantation of human pluripotent stem cell-derived cardiomyocytes is a promising preclinical therapy to restore function. Recent large animal data, however, have revealed a significant risk of engraftment arrhythmia (EA). Although transient, the risk posed by EA presents a barrier to clinical translation. We hypothesized that clinically approved antiarrhythmic drugs can prevent EA-related mortality as well as suppress tachycardia and arrhythmia burden. This study uses a porcine model to provide proof-of-concept evidence that a combination of amiodarone and ivabradine can effectively suppress EA. None of the nine treated subjects experienced the primary endpoint of cardiac death, unstable EA, or heart failure compared with five out of eight (62.5%) in the control cohort (hazard ratio = 0.00; 95% confidence interval: 0–0.297; p = 0.002). Pharmacologic treatment of EA may be a viable strategy to improve safety and allow further clinical development of cardiac remuscularization therapy.

## Highlights

• EA arises after hESC-CM transplantation in infarcted pigs
• Combination pharmacotherapy prevents EA-related mortality and morbidity
• Amiodarone and ivabradine significantly suppresses tachycardia and arrythmia burden
• EA is polymorphic and may be due to interaction with intramural Purkinje fibers

## Teaser

Potentially fatal engraftment arrhythmia (EA) arises after hESC-CM transplantation in infarcted pigs. Nakamura and colleagues present proof-of-concept evidence that a combination of amiodarone and ivabradine can effectively prevent EA-related mortality and suppresses tachycardia and arrhythmia burden. Thus, pharmacologic suppression of EA may be a viable strategy to improve safety and allow further clinical development of cardiac remuscularization therapy.

## Introduction

Ischemic heart disease, including myocardial infarction (MI) and heart failure, remains the leading cause of death in the United States and around the world. Approximately one billion cardiomyocytes are permanently lost during MI and an increasing proportion of MI survivors—an estimated 20% to 30% (Velagaleti et al., 2008)—later develop heart failure. Current treatments can slow the initiation and progression of heart failure, but none replaces lost myocardium, short of orthotopic heart transplantation, which remains restricted in availability and indication (Nakamura and Murry, 2019). Human pluripotent stem cells ([hPSCs], comprising embryonic stem cells [ESCs] and their reprogrammed cousins, induced pluripotent stem cells [iPSCs]) are a renewable source of cardiomyocytes (CMs). Transplantation of hPSC-derived cardiomyocytes (hPSC-CMs) into infarcted myocardium of small animals—mice, rats, and guinea pigs—has shown stable engraftment (Caspi et al., 2007; Laflamme et al., 2007; Shiba et al., 2012, 2014; van Laake et al., 2007). More recently, our group and others have shown remuscularization and functional benefit in infarcted non-human primates (NHPs) following transplantation of pluripotent stem cell-derived cardiomyocytes (Chong et al., 2014; Liu et al., 2018; Shiba et al., 2016). In addition to functional remuscularization, the human graft vascularizes and electromechanically couples with the host myocardium within 1 month post-transplant and remains durable up to 3 months, the longest time tested.

Although no arrhythmias were observed in smaller animals, we and others consistently observe ventricular arrhythmias following hPSC-CM transplantation in NHPs (Chong et al., 2014; Liu et al., 2018; Shiba et al., 2016) and pigs (Romagnuolo et al., 2019), which we have called “engraftment arrhythmias” (EAs). EAs are generally transient, occurring within a week of transplantation and typically resolve spontaneously after approximately 1 month. Based on electrical mapping, overdrive pacing, and cardioversion studies, EAs appear to originate focally in the graft or peri-graft myocardium and function as automatous foci rather than reentrant pathways (Liu et al., 2018; Romagnuolo et al., 2019). Although EA is reasonably well tolerated in NHPs, the Laflamme group (Romagnuolo et al., 2019) reported that EA can be lethal in pigs. For this reason, EA has emerged as the biggest impediment to the clinical translation of human cardiomyocyte transplantation (Eschenhagen et al., 2017).

We hypothesized that the risk of EA may be mitigated by treatment with clinically available antiarrhythmic drugs. Because the pig shows heightened sensitivity to EAs, is a well-established model in cardiovascular research (Lelovas et al., 2014) and cell therapy (van der Spoel et al., 2011), whose larger heart permits the use of percutaneous delivery catheters, we chose to test the hypothesis in this large animal model. In the first phase of our study, we screened a panel of seven antiarrhythmic agents. The broad-acting (class III) antiarrhythmic amiodarone and the pacemaker inhibitor (class 0) ivabradine emerged independently as the most promising agents for control of rhythm and rate, respectively. We therefore performed a second phase to test the effect of combined amiodarone and ivabradine treatment. We found that this regimen reduced sudden cardiac death, as well as suppressed tachycardia and arrhythmia.

## Results

### Clinical history of EA

**Figure 1:**
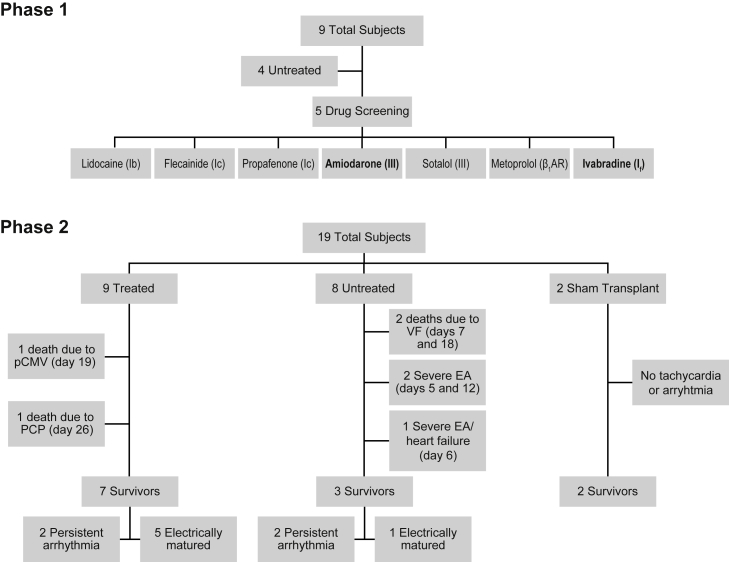
Flowchart of the study design Phase 1 consisted of nine total subjects, four untreated with any antiarrhythmic to study the natural history of EA and five used to screen seven candidate antiarrhythmic agents. Amiodarone and ivabradine were found to have promising signals of effect and advanced for further study. Phase 2 consisted of 19 total subjects: 9 treated with amiodarone and ivabradine and 8 untreated following hESC-CM transplantation, and 2 untreated following sham transplantation.

**Figure 2:**
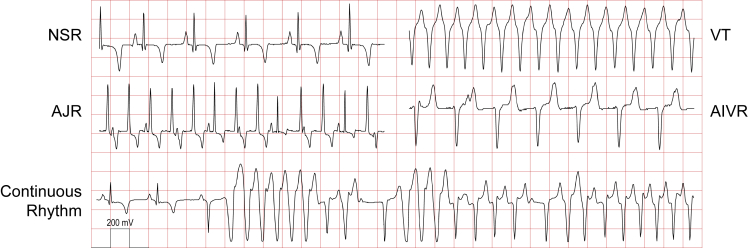
Variable morphologies of EA in a single minipig Examples of normal sinus rhythm (NSR) and three morphologies of EA resembling accelerated junctional rhythm (AJR), ventricular tachycardia (VT), and accelerated idioventricular rhythm (AIVR) were observed in a single untreated subject (12). Note the variation in rate, electrical axis, and QRS duration. A continuous rhythm recording below exhibits polymorphic EA with QRS complexes varying in rate, duration, and electrical axis. No sustained arrythmias were noted in the sham transplant controls. One box vertical 200 mV, horizontal 0.2 s.

A flow chart for all subjects in the study is shown in Figure 1 and clinical summaries are provided in Table 1. No significant arrhythmias were noted in the two untreated sham transplant control subjects (9 and 10) that underwent MI and percutaneous intracardiac injection of vehicle. All subjects that received human cardiomyocyte grafts developed EA between 2 and 6 days following cell transplantation. Initiation of EA was characterized by salvos of non-sustained ventricular tachycardia (VT), and this typically progressed to periods of sustained VT with rates ranging from 110 to 250 bpm (Figure 2). The VT was often polymorphic, with the same subject showing different electrical axes and both wide and narrow complex tachycardia at different times. In four of the eight untreated subjects, EA was either fatal or necessitated euthanasia due to a prespecified endpoint of unstable tachycardia (defined as sustained heart rate >350 bpm). In one additional untreated case (subject 12), acute heart failure was noted clinically after a week of EA at a persistent rate of 300 bpm and, based on recommendations from veterinary staff, the subject was euthanized. Signs of heart failure were subsequently confirmed on necropsy. In all other cases, EA was noted with a rapid acceleration to >350 bpm (subjects 11 and 14) and, in two cases, deterioration to ventricular fibrillation (VF) prior to euthanasia (subjects 1 and 2) (Table 1). Three out of four arrhythmic endpoints occurred within the first 3 days of developing EA, and they occurred when tachyarrhythmia was nearly constant. Mean heart rate peaked at day 8 post-transplantation and thereafter began to decline, whereas the arrhythmia burden plateaued from days 8 to 16 and began to normalize thereafter. Of the three survivors in the untreated cohort, two did not normalize rhythm and experienced, on average, 42% arrhythmia burden at the end of study (subjects 17 and 19). The single subject in the untreated cohort that normalized heart rate and rhythm did so on post-transplant day 25 (subject 13).

**Table 1:** Characteristics of study subjects and clinical history

Subject	Age (days)	Weight (kg)	MI	Cell line	Approach	hESC-CM cTnT (%)	hESC-CM viability (%)	Infarct size (%)^a^	Graft size (%)^b^	Graft mass (g)	LV mass (g)	HR (bpm)		Arrhythmia burden (% of time)		Outcome
												Day 7	Day 30	Day 7	Day 30	
**Treated**																

3	231	34.7	yes	H7	Surg	98	89	6.8	5.4	0.5	125.9	73	N/A	2.0	N/A	euthanasia, day 26 (PCP)
4	227	32.0	yes	H7	Perc	98	89	8.2	7.3	0.8	137.9	93	94	86.0	97.8	survival
5	236	33.0	yes	RUES2	Perc	91	89	7.1	9.0	0.5	84.0	163	81	25.6	0.5	survival
6	231	33.4	yes	RUES2	Perc	91	90	10.1	1.4	0.1	94.2	100	N/A	47.1	N/A	euthanasia, day 19 (pCMV)
7	230	37.0	yes	RUES2	Perc	91	93	9.6	0.3	0.0	142.7	89	79	45.8	28.2	survival
8	299	33.5	yes	RUES2	Perc	86	88	15.9	3.4	0.8	153.3	73	77	67.6	4.7	survival
15	271	33.0	yes	RUES2	Perc	89	88	10.9	4.2	0.5	113.4	79	76	35.4	44.9	survival
16	263	34.0	yes	RUES2	Perc	88	85	14.6	0.7	0.1	136.7	74	69	43.9	7.6	survival
18	380	32.5	yes	RUES2	Perc	94	75	9.3	3.7	0.5	144.5	69	78	1.6	1.6	survival
Avg ± SEM	263.1 ± 16.8	33.7 ± 0.5				92 ± 1	87 ± 2	10.3 ± 1	3.9 ± 1	0.4 ± 0.1	125.8 ± 8	90 ± 10	79 ± 3	39.5 ± 9.2	26.5 ± 13.4	

**Untreated**																

1	267	32.0	no	H7	Surg	98	88	N/A	N/A	0.3	75.10	328	N/A	100.0	N/A	primary endpoint day 7 (VF)
2	314	32.0	yes	H7	Surg	98	90	5.8	5.6	0.3	92.80	120	N/A	62.7	N/A	primary endpoint day 19 (VF)
11	284	35.0	yes	RUES2	Perc	86	90	13.2	1.0	0.2	134.9	N/A	N/A	N/A	N/A	primary endpoint day 5 (EA)
12	283	33.5	yes	RUES2	Perc	82	87	9.7	1.1	0.1	112.4	166	N/A	94.0	N/A	primary endpoint day 12 (EA/HF)
13	292	35.5	yes	RUES2	Perc	87	83	16.5	1.7	0.3	107.4	162	83	95.9	1.9	survival
14	270	33.0	yes	RUES2	Perc	87	90	4.6	9.1	0.4	93.2	N/A	N/A	N/A	N/A	primary endpoint day 6 (EA)
17	209	33.0	yes	RUES2	Perc	98	70	13.5	0.4	0.1	104.3	106	115	49.1	75.7	survival
19	192	33.5	yes	RUES2	Perc	92	74	5.4	3.6	0.3	139.2	98	84	71.5	51.7	survival
Avg ± SEM	263.9 ± 14.8	33.4 ± 0.4				91 ± 2	84 ± 3	9.8 ± 1.8	3.2 ± 1.2	0.2 ± 0	107.4 ± 7.6	163 ± 35	94 ± 11	78.9 ± 8.5	43.1 ± 21.7	
p value^c^	0.97	0.73				0.77	0.31	0.82	0.64	0.08	0.12	0.03	0.09	0.01	0.52	

**Sham transplant control**																

9	249	33.5	yes	N/A	Perc	N/A	N/A	–	N/A	N/A	N/A	71	68	0.8	0.0	survival
10	233	33.0	yes	N/A	Perc	N/A	N/A	–	N/A	N/A	N/A	77	69	1.2	0.8	survival
Avg ± SEM	241 ± 8	33.3 ± 0.3										74 ± 3	69 ± 1	1.0 ± 0.2	0.4 ± 0.4	

bpm, beats per minute; CK, cytokeratin; EA, engraftment arrhythmia; hESC-CM, human embryonic stem cell-derived cardiomyocytes; HF, heart failure; HR, heart rate; MI, myocardial infarction; N/A, not applicable; surg, surgery; PCP, *Pneumocystis* pneumonia; pCMV, porcine cytomegalovirus; perc, percutaneous; VF, ventricular fibrillation.

a % of left ventricle.

b % of infarct area.

c Treated versus untreated.

### Screening drugs for antiarrhythmic effects

**Figure 3:**
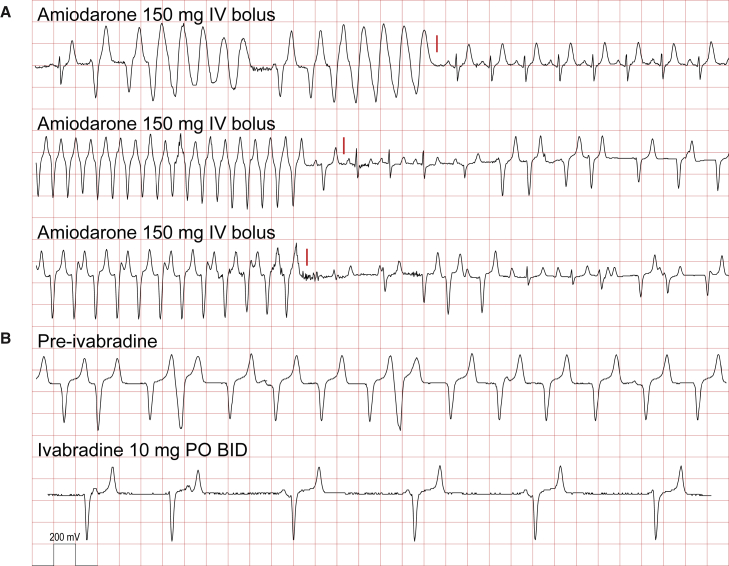
Acute effects of amiodarone and ivabradine on engraftment arrhythmia Amiodarone was effective as an intravenous bolus to cardiovert engraftment arrythmia to normal sinus or a lower heart rate transiently in three separate instances (A) (red line). Ivabradine administered orally significantly slowed EA but did not cardiovert 2 days following initiation (B). These data supported a combined amiodarone and ivabradine antiarrhythmic strategy for rhythm and rate control of EA. One box vertical 200 mV, horizontal 0.2 s.

In phase 1 of the study, we screened six canonical antiarrhythmic agents broadly targeting sodium channels, potassium channels, and β-adrenergic receptors: lidocaine (class Ib Vaughan-Williams-Singh antiarrhythmic, sodium channel inhibitor), flecainide (Ic, sodium channel inhibitor), propafenone (Ic, sodium channel inhibitor), amiodarone (III, potassium channel inhibitor), sotalol (III, potassium channel inhibitor), and metoprolol (β1-adrenergic receptor inhibitor) for effect on EA heart rate and rhythm. In addition, the funny current/HCN4 channel antagonist, ivabradine, was tested (Table S1). This series was not meant to be comprehensive but rather to rapidly identify candidate agents. Animals were brought into the laboratory while in EA, anesthetized, and the effects of short-term intravenous infusion or oral treatment of antiarrhythmic agents were studied. In three instances, intravenous amiodarone successfully cardioverted unstable EA from >350 bpm to a lower heart rate, typically including brief episodes of sinus rhythm (Figure 3A). Oral ivabradine demonstrated robust dose-dependent effects on heart rate but did not restore sinus rhythm (Figure 3B). Four of the other drugs had no significant effect in this screen (lidocaine, flecainide, sotalol, and metoprolol). Propafenone briefly reduced heart rate and restored sinus rhythm in two-drug challenges, but this drug was associated with substantial gastrointestinal toxicity and not studied further (data not shown).

### Amiodarone-ivabradine enhance survival

**Figure 4:**
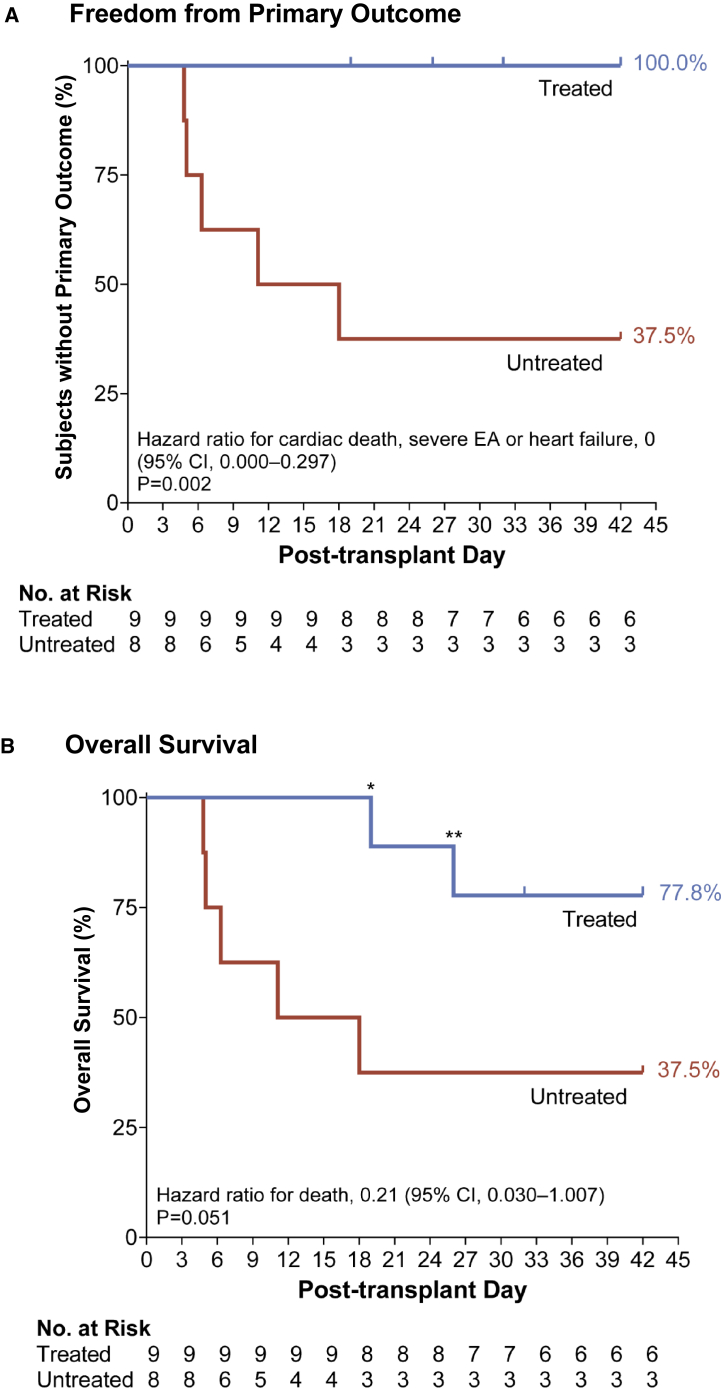
Antiarrhythmic treatment with amiodarone and ivabradine for engraftment arrhythmia in pig (A) Kaplan-Meier curve for freedom from primary outcome of cardiac death, unstable EA or heart failure was significantly improved in the treated (n = 9) compared with the untreated cohort (n = 8, p = 0.002). Tic marks on the treatment line indicate non-cardiac death due to opportunistic infection (days 19 and 26) or a planned euthanasia (day 30). (B) The Kaplan-Meier curve for overall survival shows statistically borderline improvement in the treated compared with the untreated cohort (p = 0.051). Each subject is an independent experiment. ^∗^Death due to *Pneumocystis* pneumonia. ^∗∗^Death due to porcine cytomegalovirus. CI, 95% confidence interval; No., number.

Given their distinct mechanisms of action and complementary effects on heart rate and rhythm, we formally tested the hypothesis that amiodarone along with ivabradine would reduce a combined primary endpoint of cardiac death, unstable EA >350 bpm, and heart failure in phase 2 of the study. A total of nine treated, eight untreated, and two sham transplant subjects were enrolled in the study with similar baseline and cell transplantation characteristics (Table 1). As detailed in the experimental procedures, treated animals received bolus and maintenance doses of amiodarone, and ivabradine was given as needed to keep heart rates <150 bpm. All treated subjects survived without the primary cardiac endpoint compared with 3/8 (37.5%) of untreated subjects (Figure 4A). The hazard ratio of the primary endpoint was 0.000 (95% CI: 0.000–0.297; p = 0.002) with antiarrhythmic treatment. Of note, two of the treated subjects (3 and 6) experienced non-cardiac death at post-transplant days 26 and 19 due to immunosuppression-related complications (*Pneumocystis* pneumonia and porcine cytomegalovirus, respectively). Intention-to-treat analysis of overall survival also favored the treated cohort with hazard ratio of 0.212 (95% CI: 0.030–1.007; p = 0.05) (Figure 4B).

### Suppression of tachycardia and arrhythmia burden

**Figure 5:**
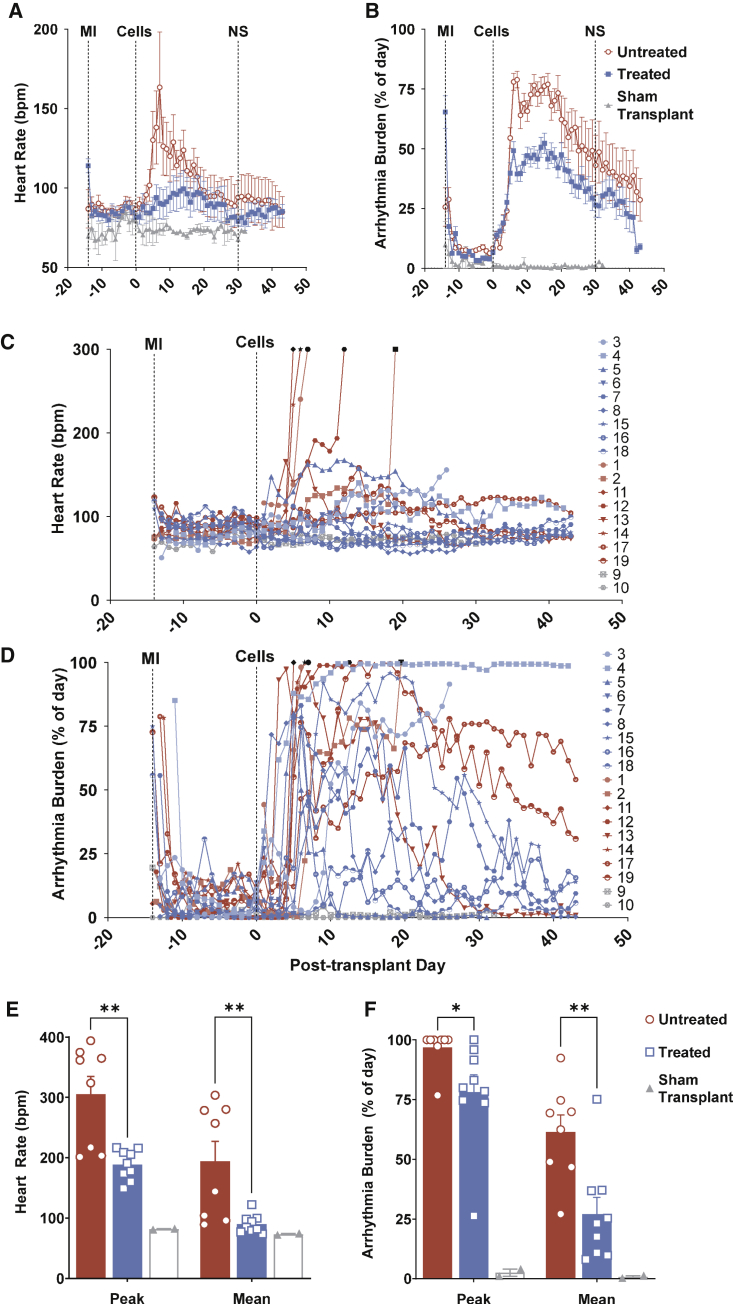
Effect of antiarrhythmic treatment on heart rate and arrhythmia burden Pooled daily average heart rate (A) and pooled daily average arrhythmia burden (B) in treated (n = 9, blue) compared with untreated (n = 8, red) cohorts. The difference in heart rate or arrhythmia burden between treated and untreated cohorts was not significant (NS) by day 30 post-transplantation. Sham transplant (n = 2, gray) did not induce tachycardia or arrythmia. Data are represented as mean ± SEM and each subject is an independent experiment. Subject-level averaged daily heart rate (C) and arrhythmia burden (D) for antiarrhythmic treated (blue), untreated (red), and sham transplant (gray). Unexpected death or euthanasia are denoted by black symbols. Overall peak and mean daily heart rate (E) and overall peak and mean daily arrythmia burden (F) were significantly reduced in treated (blue) compared with untreated (red) cohorts. No tachycardia or arrhythmias were noted in the sham transplant control cohort. ^∗^p ≤ 0.05, ^∗∗^p ≤ 0.01.

Pooled and individual subject-level data of heart rate and arrhythmia burden are provided in Figures 5A–5D, respectively. The average heart rate was significantly lower with antiarrhythmic treatment compared with no treatment. Mean heart rates peaked at post-transplantation day 7 in untreated animals at 163 ± 35 bpm, versus average heart rates of 90 ± 10 bpm in the treated cohort (p = 0.03) (Table 1; Figure 5A). Heart rate in the treated animals was not significantly different than the normal resting heart rate before MI and transplant (84 ± 1 bpm, p = 0.21). Following transplantation, peak daily heart rate for the study duration averaged 305 ± 29 bpm in untreated animals, whereas treatment significantly attenuated peak heart rate to 185 ± 9 bpm (p = 0.001) (Figure 5E). We defined arrhythmia burden as the percentage of the day spent in arrhythmia. Treatment reduced peak arrhythmia burden from 96.8% ± 2.9% to 78.1% ± 7.2% (p = 0.04), and the average daily burden was reduced from 61.4 ± 7.1% to 27.1 ± 7.0% (p = 0.004) (Figure 5F). No differences in heart rate or arrhythmia burden were noted at post-transplant day 30, as the majority of arrhythmia had resolved irrespective of treatment (Figures 5A and 5B) (p = 0.09 and p = 0.52, respectively).

Antiarrhythmic treatment was safely discontinued between days 24 and 34 in all treated subjects that achieved electrical maturation without recrudescence of arrhythmia (Figure 5). Two treated and two untreated subjects (3 and 4 and 17 and 19, respectively) failed to mature electrically and exhibited significant arrhythmia at the end of study. In these four animals, heart rates were well controlled irrespective of treatment, and they survived until the study's completion. Average serum amiodarone was sub-therapeutic at 0.42 ± 0.12 μg/mL within 1 week of discontinuation (Figure S2).

### Percutaneous delivery of hESC-CMs in the infarcted porcine model

Catheter-based endocardial delivery of human ESC (hESC)-CMs was safe and effective in remuscularizing the infarcted porcine heart (Figure S3). No significant differences in myocardial infarct or cardiomyocyte graft sizes were observed between the treatment groups. The average infarct size for the treated and untreated cohorts were comparable at 10.3% ± 1% and 9.8% ± 1.8% of the left ventricle, respectively (p = 0.82). Graft size relative to infarct size was also comparable at 3.9% ± 1% and 3.2% ± 1.2% for treated and untreated subjects, respectively (p = 0.64). Delivery of hESC-CM successfully targeted the peri-infarct border zone and central ischemic regions as intended and resulted in discrete hPSC-CM grafts transplanted into host myocardium, as reported previously (Chong et al., 2014; Liu et al., 2018; Romagnuolo et al., 2019; Shiba et al., 2016). All grafts localized to the anterior, antero-septal, and antero-lateral walls and, as previously reported in pig (Romagnuolo et al., 2019), appeared structurally immature at early time points before 2 weeks post-transplantation with increasing maturity up to the end of study.

### Graft interaction with the host Purkinje conduction system

**Figure 6:**
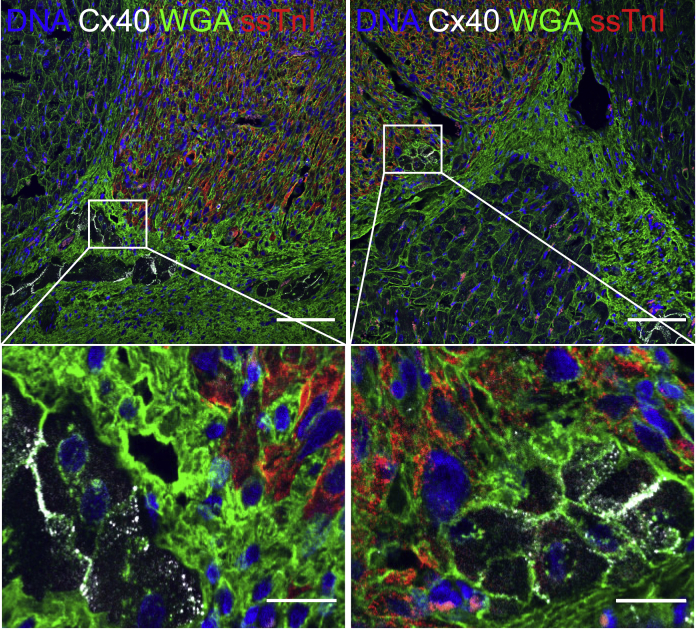
Transplanted hESC-CM grafts interact with a diffuse Purkinje conduction system in the porcine myocardium An hPSC-cardiomyocyte graft 2 weeks post-transplantation, marked by human-specific slow skeletal cardiac troponin I (ssTnI) (red) (Bedada et al., 2014), interacts with Cx40-positive (white) Purkinje fibers. Wheat germ agglutinin (WGA) (green) delineates cardiac sarcolemma. Purkinje-transitional cell-graft (left panel) and direct Purkinje-graft (right panel) interactions are observed. Sale bars, 100 or 20 μm (magnified).

The narrow complex tachycardia that resembles accelerated junctional rhythm (Figure 2) was not observed in our previous NHP studies (Chong et al., 2014; Liu et al., 2018) but was common in the minipig. Pigs are known to have an extensive Purkinje fiber network that extends transmurally throughout the ventricular myocardium, whereas in macaques and humans the Purkinje network is subendocardial (Garcia-Bustos et al., 2017; Panescu et al., 2014). We hypothesized that narrow complex VT resulted from graft automaticity conducting through intramural Purkinje fibers and propagating to the rest of the ventricle. Histology confirmed the mesh-like network of intramural Purkinje fibers throughout the minipig left ventricle (Figure S4A; Video S1). There were multiple examples of hESC-CM grafts in direct contact with these intramural branches of the Purkinje system (Figure 6; Video S2). We used connexin 40 (Cx40) immunostaining to specifically stain Purkinje fiber gap junctions (Garcia-Bustos et al., 2017; Pallante et al., 2010), and confirmed their identity by their reduced myofibril content and the absence of T tubules (Figure S4B). This supports the hypothesis that the pig's unique Purkinje network anatomy contributes to narrow complex EA.

Video S1. Purkinje fibers are distributed in a mesh-like network throughout the native porcine myocardium Confocal z stack of 28 images, z step size 0.7 μm. Blue, DNA (Hoechst); green, WGA; red, F-actin (phalloidin); white, CX40.

Video S2. hESC-cardiomyocytes marked by slow skeletal troponin I (ssTnI) interact with connexin 40+ Purkinje fibers Confocal z stack of 56 images, z step size 1 μm. Blue, DNA; green, F-actin; red, ssTnI; white, CX40.

## Discussion

Intramyocardial transplantation of hPSC-CMs is a promising strategy to remuscularize the infarcted heart and restore function (Nakamura and Murry, 2019). Such a therapy to prevent and treat heart failure would be a seminal advance in addressing a large unmet clinical need. Studies in large animals have demonstrated long-term efficacy but also defined a significant safety signal of generally transient but potentially fatal arrhythmias. As demonstrated in earlier studies (Liu et al., 2018; Romagnuolo et al., 2019; Shiba et al., 2016), EA is a predictable complication of cardiac remuscularization therapy for MI (Yu et al., 2019). In the NHPs, EA typically presents as a wide complex tachycardia with a variable electrical axis (Chong et al., 2014; Liu et al., 2018), and this was reproduced in the minipig recently by the Laflamme laboratory (Romagnuolo et al., 2019). Here, we further describe EA as polymorphic and interpret the changes in electrical axis as ectopy originating from different graft foci. Interestingly, in the pig we also observed a narrow complex VT that alternated with a wide complex tachycardia, a pattern not seen in the NHPs. Histology of native and grafted porcine myocardium supports the hypothesis that the wide complex beats originate from grafts interacting with the working cardiac myocytes with slow conduction, and that the narrow complex beats originate when grafts interact with the intramural Purkinje fibers that diffusely permeate the porcine heart (Garcia-Bustos et al., 2017; Panescu et al., 2014).

All 17 subjects transplanted with 500 × 10^6^ hESC-CMs demonstrated significant burden of arrhythmia that, while typically transient, was associated with high mortality in pigs. We observed higher morbidity and mortality related to EA than the recent study by Laflamme and colleagues (Romagnuolo et al., 2019), perhaps reflecting differences in our animal model including the use of Yucatan minipigs, percutaneous cell delivery, or our cell product. Our experience with this model suggests two primary mechanisms of cardiac morbidity. Firstly, rapid EA >350 bpm often degenerates to fatal VF and, secondly, heart failure commonly ensues in pigs with chronic tachycardia >230 bpm (Chow et al., 1990). Consequently, our primary endpoint included these parameters to limit excessive mortality in our antiarrhythmic trial.

Combined antiarrhythmic treatment with baseline amiodarone and adjunctive ivabradine safely prevented the combined primary endpoint of cardiac death, unstable EA, and heart failure in all treated subjects, indicating that the risk of EA may be mitigated through pharmacology. Treatment was associated with significantly decreased peak tachycardia and arrhythmia. Once subjects experienced sustained improvement in arrhythmia burden, termed electrical maturation, antiarrhythmic therapy was successfully withdrawn in all subjects. Thus, short-term amiodarone and ivabradine treatment promoted electrical stability until the grafts became less arrhythmogenic.

The mechanism of benefit for our antiarrhythmic treatment may be related to suppression of automaticity, reducing both heart rate and arrhythmia burden. The drugs were particularly beneficial during the early phase of EA, which carries the greatest risk of deterioration to VF. Electrophysiological studies performed by our group and the Laflamme laboratory in NHPs (Liu et al., 2018) and pig (Romagnuolo et al., 2019), respectively, suggests that the etiology of EA is increased focal automaticity, rather than macro-reentry typically observed with clinical VT (Josephson et al., 1984). In our study, cardioversion was unsuccessful in terminating EA. As EA became unstable in the untreated animals, heart rates rapidly accelerated to >350 bpm, and we cannot exclude the possibility that this escalation could have a distinct mechanism, e.g., automaticity leading to reentry. This may explain why treatment successfully suppressed unstable and fatal arrhythmias but was unable to prevent EA altogether.

The efficacy of ivabradine to rate control EA suggests that its pharmacologic target, the I_f_ current carried by the HCN4 channel, which is highly expressed in immature cardiomyocytes and hPSC-CMs (Karbassi et al., 2020), may be an important mediator. Ivabradine, by itself, never abrogated EA, suggesting that, while the I_f_ current can accelerate the rate of EAs, I_f_ is not the current responsible for generating the ventricular automaticity triggered by engraftment. In contrast, amiodarone reduced the burden of EA chronically and clearly restored sinus rhythm in some acute infusion experiments (Figure 3). Although classified principally as a K^+^ channel blocker (class III), amiodarone is well known also to antagonize Na^+^ channels, Ca^2+^ channels, and β-adrenergic receptors (Waks and Zimetbaum, 2017). Thus, it is difficult to gain insights into the mechanism of EA from amiodarone's efficacy. The disappearance of EA coincides with maturation of the stem cell-derived graft (Chong et al., 2014; Kadota et al., 2017; Liu et al., 2018), and we with others have hypothesized that the window of arrhythmogenicity may reflect a period of *in vivo* graft maturation before reaching a state more similar to host myocardium (Guo and Pu, 2020; Ichimura et al., 2020; Kannan and Kwon, 2020; Karbassi et al., 2020; Marchiano et al., 2019; Maroli and Braun, 2020). Additional strategies such as promoting maturation before transplantation, gene editing, and modulating host/cell interaction may provide additional means of arrhythmia control and a comprehensive protocol invoking multiple complementary mechanisms of action may ultimately be necessary to ensure safety. Further investigation of the etiology of EA would be accelerated by the development of higher throughput *in vivo*, *ex vivo*, *in vitro*, and/or *in silico* platforms to perform genetic, pharmacological, electrophysiological, and/or modeling studies before phenotyping in large animal models.

EA is the most significant barrier to clinical translation of cardiac remuscularization therapy. The natural history of EA emerging from the NHPs and more recent porcine data suggests that, once EA resolves, there is low risk for further arrhythmia. This study provides a proof-of-concept that clinically relevant antiarrhythmic drug treatment can successfully suppress fatal arrhythmias and control tachycardia to achieve electrical quiescence. This could be an important tool toward reaching an acceptable safety profile for clinical development.

While this study demonstrates that EA is responsive to pharmacologic suppression, there are several limitations. Notably, our proposed regimen did not completely abrogate EA and several treated subjects experienced significant, but not fatal, arrhythmia. As such, adjunctive methods may be desirable to further mitigate risk from EA to patients. Possible strategies may include promoting maturation and modification of hESC-CMs as well as dosing and delivery protocols to be less arrhythmogenic. This study would also benefit from longer follow-up to establish the long-term effectiveness of EA mitigation. We did not randomize enrollment of animals or assess whether sex is a biological variable. Although we took pains to administer clinically relevant doses of amiodarone and ivabradine, we cannot exclude the possibility that EA in itself is dependent on the dose of cells transplanted. The dose utilized is comparable with that utilized to demonstrate long-term function benefit in NHPs (500 × 10^6^ versus 750 × 10^6^ hESC-CMs, respectively) (Liu et al., 2018), but dosing studies have not been reported. Future studies will also ideally include functional endpoints to determine mechanical efficacy with background guideline-directed medical therapy, such as inhibitors of the renin-angiotensin-aldosterone and β-adrenergic systems.

In this study utilizing a porcine infarction model of cardiac remuscularization therapy, EA was universally observed and associated with significant mortality. Chronic amiodarone treatment combined with adjunctive ivabradine successfully prevented the combined primary endpoint of cardiac death, unstable EA, and heart failure. Overall survival was significantly improved with antiarrhythmic treatment and associated with heart rate and rhythm control. The mechanisms of EA remain incompletely understood and merit concerted scientific inquiry.

## Experimental procedures

### hESC-CM production

These studies were approved by the University of Washington Stem Cell Research Oversight Committee. Two lines of hESCs were used in this study. Initial subjects received H7 (WiCell)-derived cardiomyocytes that were cultured, expanded, and differentiated in suspension culture format by collaborators at the Center for Applied Technology Development at the City of Hope in California, all as described previously (Chen et al., 2015; Chong et al., 2014; Liu et al., 2018). Most subjects received RUES2 (Rockefeller University)-derived cardiomyocytes produced in our laboratory in stirred suspension culture format. In brief, RUES2 hESCs were cultured to form aggregates and were expanded in commercially available medium (Essential 8, Gibco). For cardiac differentiation, suspension-adapted pluripotent aggregates were induced to differentiate in RPMI-1640, MCDB-131, or M199 supplemented with B-27 (all from Gibco) or serum albumin, by timed use of small-molecule GSK 3 inhibitor, CHIR 99021 (days 0 to 1), and Wnt/β-catenin signal pathway inhibitors (days 2 to 4) (Tocris). Twenty-four hours before cryopreservation, RUES2 hESC-CMs were heat shocked to enhance their survival after harvest, cryopreservation, thaw, and transplantation. Cardiomyocyte aggregates were dissociated by treatment with Liberase TH (Fisher) and TrypLE (Gibco) and were cryopreserved in CryoStor CS10 (STEMCELL Technologies) supplemented with 10 μM Y-27632 (STEMCELL Technologies) using a controlled-rate liquid nitrogen freezer. Approximately 3 h before transplantation, cryopreserved hESC-CMs were removed from cryogenic storage (−150°C to −196°C) and thawed in a 37°C water bath (2 min ± 30 s). RPMI-1640 supplemented with B-27 and ≥200 Kunitz units/mL DNase I (Millipore) was added to the cell suspension to dilute the cryopreservation media. Subsequent wash steps were done using RPMI-1640 basal medium in progressively smaller volumes to concentrate the cell suspension. For the last centrifugation step, the cell pellet was resuspended in a sufficient volume of RPMI-1640 to achieve a target cell density for injection of ∼3 × 10^9^ cells/mL in 1.6 mL. The final volume of the cell suspension was determined by the results of a count sampled before the final centrifugation step. Cell counts were performed as described previously to achieve a final total dose of 500 × 10^6^ live cells per transplant.

### Study design

The objective of this study was to identify a pharmacological regimen to attenuate arrhythmias following cardiac remuscularization therapy. This study was designed in two phases: the first to observe the natural history of EA in the minipig model and screen various antiarrhythmic agents for possible efficacy, and the second to rigorously test for efficacy of selected candidates (Figure 1). All protocols were approved and conducted in accordance with the University of Washington (UW) Office of Animal Welfare and the Institutional Animal Care and Use Committee. All subjects were 30–40 kg castrated male Yucatan minipigs between 6 and 13 months of age (Premier BioSource). In phase 1, nine subjects underwent cardiac remuscularization therapy with 500 × 10^6^ hESC-CMs delivered by direct surgical trans-epicardial injections, or later by percutaneous trans-endocardial injections (Table 1). The first four subjects (one non-infarcted and three infarcted) were followed to learn the natural history of EA and establish clinical endpoints and parameters for the phase 2 trial. The subsequent five subjects underwent systematic dosing with antiarrhythmic agents with continuous electrocardiography (ECG) monitoring to determine effect on rhythm and rate (Table S1, supplemental information). In brief, subjects were administered serial trials of antiarrhythmics and observed for acute response by continuous ECG monitoring. All agents were tested in at least two subjects. Details are provided in the supplemental information.

Among the nine subjects in phase 1, we observed high mortality, with six out of nine experiencing VF or tachycardia-induced heart failure requiring euthanasia. VF typically occurred within the first week of transplantation and following frequent episodes of unstable EA >350 bpm, and tachycardia-induced heart failure requiring euthanasia was characterized by chronically elevated heart rates >150 bpm for 1–2 weeks.

In phase 2, we conducted a two-drug antiarrhythmic study with amiodarone and ivabradine, enrolling an additional 17 subjects (9 treated, 8 untreated) that underwent MI and percutaneous transplantation with hESC-CMs at 2 weeks post-MI. Two additional subjects underwent MI with sham vehicle injection to serve as sham transplant controls (Figures 1 and S1). The primary endpoint was prespecified as combined cardiac death (either spontaneous death from arrhythmia or heart failure, or clinically directed euthanasia necessitated by sustained tachycardia >350 bpm or signs of heart failure). Prespecified secondary endpoints were suppression of tachycardia, percent time in arrhythmia (arrhythmia burden) and resolution of arrhythmia, termed “electrical maturation” and defined as arrhythmia burden <25% for 48 consecutive hours. Antiarrhythmic therapy was discontinued after electrical maturation or at post-transplantation day 30, whichever was earlier. To prevent tachycardia-induced cardiomyopathy, we titrated ivabradine treatment to maintain target heart rate <150 bpm. Based on early experience that tachycardia >350 bpm often degenerated to VF, subjects were euthanized humanely if heart rates >350 bpm were reached. Continuous telemetric ECG was monitored for 8 weeks in total (2 weeks post-MI and 6 weeks post-transplantation). Of note, subjects 1 and 2 (untreated) and 3 and 4 (treated) received H7 hESC-CMs and subjects 1–3 were transplanted surgically before adopting percutaneous delivery. Subject 5 exhibited early electrical maturation on day 12 and was euthanized on day 31 before extending the study duration to 6 weeks post-transplantation for extended treatment washout and monitoring for all subjects.

### Cardiac remuscularization therapy

MI was induced percutaneously 2 weeks before cell transplantation (supplemental information). Cell transplantation for our three initial subjects (1–3) was performed by direct trans-epicardial injection into the peri-infarction region as previously described for NHPs with minor modification (Liu et al., 2018) and described in the supplemental information. All subsequent subjects (4–19) received cell transplantation via percutaneous trans-endocardial injection using the NOGA-MyoStar platform (BioSense Webster). In brief, trans-epicardial injection via partial median sternotomy was performed to expose the infarcted anterior left ventricle. Purse-string sutures were preplaced at five discrete locations subtended by the LAD, targeting the central ischemic region (2/5) and lateral border zones identified by gross inspection and adjudicated by consensus of the surgical team. After cinching the purse-string tightly around the needle, three injections of 100 μL each were performed by partial withdrawal and lateral repositioning, for a total of 15 injections to deliver a total dose of 500 × 10^6^ hESC-CMs. Percutaneous trans-endocardial injection using the NOGA-MyoStar platform (BioSense Webster) was performed by first mapping the infarct region in the left ventricle, and then deliver 16 discrete endocardial injections of 100 μL each for a total dose of 500 × 10^6^ hESC-CMs. Injections were only performed with excellent location and loop stability, ST-segment elevation, and the presence of premature ventricular contraction with needle insertion in an appropriate location by electroanatomical map and unipolar voltage. Two-thirds of injections were placed into the peri-infarct border zone defined by a unipolar voltage of 5–7.5 mV, and the remaining one-third into the central ischemic region, defined as unipolar voltage of <5 mV. Two subjects (9 and 10) were infarcted as per protocol but received sham injections of RPMI-1640 vehicle without cells to serve as sham transplant controls.

### Immunosuppression therapy

All three cohorts received a three-drug immunosuppression regimen to prevent xenograft rejection, as described previously with modification (Liu et al., 2018). For our initial regimen (subjects 1–6), 5 days before cell transplantation, oral cyclosporine A was started to maintain a serum trough level of >400 ng/mL (approximately 250–1,000 mg twice daily) for the duration of the study. Two days before transplantation, oral methylprednisolone was started at 3 mg/kg for 2 weeks and then titrated down to 1.5 mg/kg for the remainder of the study. On the day of transplantation, Abatacept (CTLA4-Ig, Bristol-Myers Squibb), 12.5 mg/kg, was administered intravenously and dosed every 2 weeks thereafter. Due to complications related to immunosuppression (principally porcine cytomegalovirus and *Pneumocystis* pneumonia), the cyclosporine A trough level was decreased to >300 ng/mL and the methylprednisolone reduced to 1.0 mg/kg for subjects 7–19 without histologic evidence of rejection. Prophylactic oral cephalexin was administered for all subjects to prevent infection of the indwelling central venous catheter. Prophylactic sulfamethoxazole/trimethoprim was added after subject 3 developed *Pneumocystis* pneumonia. Prophylactic valganciclovir and probiotics were added after activation of endogenous porcine cytomegalovirus (CMV) was found in subject 6. Further complications related to CMV were not observed in subsequent subjects with prophylaxis.

### Antiarrhythmic treatment

The treated cohort was loaded with oral amiodarone 1,000–1,200 mg orally twice daily starting 7 days before cell transplantation followed by a maintenance dose of 400–1,000 mg orally twice daily to maintain a steady-state plasma level of 1.5–4.0 μg/mL (Figure S2). Ivabradine was started at 2.5 mg orally twice daily when sustained tachycardia reached ≥150 bpm and titrated every 3 days up to 15 mg twice daily for a goal heart rate of <125 bpm. All but one subject (1) required adjunctive ivabradine for additional heart rate control. Antiarrhythmics were discontinued after electrical maturation was achieved or at post-transplantation day 30, whichever was earlier, to allow for treatment washout and assess for recrudescence of arrhythmia. All subjects tolerated the antiarrhythmic regimen without complication. Untreated and sham transplant control subjects did not receive antiarrhythmic agents following the MI procedure, but otherwise received all immunosuppression and standard care.

### Amiodarone drug monitoring

Liquid chromatography-mass spectrometry was used to monitor steady-state serum levels of amiodarone in the minipig model and guide oral dosing to ensure efficacy and avoid dose-related toxicity. A target serum level of 1.5–4.0 μg/mL was extrapolated from previous human pharmacokinetic studies (Mostow et al., 1984; Staubli et al., 1983). Elimination kinetics after discontinuation of oral amiodarone therapy were also studied by obtaining weekly trough concentrations in six subjects (6, 7, 8, 13, 14, and 16) (Figure S2).

### ECG analysis

Telemetric ECG was continuously monitored in real time from the time of MI to detect the primary endpoint of cardiac death or unstable EA. Automated quantification of heart rate and arrhythmia burden was performed offline by a board-certified cardiologist using the ecgAUTO 3.3.5.10 software package (EMKA Technologies). Arrhythmia was defined as an ectopic beat (e.g., premature ventricular contraction) or rhythm (e.g., idioventricular rhythm, VT). EA was typically observed as sustained and non-sustained ventricular tachyarrhythmia of varying rates and morphologies, but also included slow and narrow complex ectopic rhythms (Figure 2). Heart rate and arrhythmia burden were quantified continuously for phase 1 subjects. For phase 2 subjects, two continuous minutes every 5 min (40% of total rhythm was counted) was analyzed and presented as daily averages.

### Statistical analysis

Statistical analyses and graphing were performed using Prism 8.4.2 software (GraphPad) and Stata 15 (StataCorp, College Station, TX). Data are presented as mean ± standard error of the mean (SEM). Comparisons were performed using the Mann-Whitney test with significance threshold of p < 0.05. The sample size to demonstrate a difference in mortality rate of 67% (untreated group) versus 0% (treatment group), with alpha = 0.05 and 90% power, was estimated to be 8 per cohort. Kaplan-Meier plots show survival curves for the primary endpoint of cardiac death, unstable EA or heart failure, and for all-cause mortality. Cox proportional regression models were used to estimate the hazard ratio between the two treatment groups for the primary outcome and for mortality. Significance is based on the likelihood ratio test and confidence intervals on hazard ratio were computed by inverting the likelihood test, based on varying the offset term in the stcox procedure in Stata.

### Histologic analysis

Histological studies were carried out as detailed previously with modification (Chong et al., 2014; Liu et al., 2018). In brief, paraformaldehyde-fixed hearts were dissected to remove the atria and right ventricle before short-axis cross-sections were cut at 2.5 mm intervals. The weights of the whole heart, left ventricle, and each slice were obtained before further partition into tissue cassettes. The tissue then was processed, embedded in paraffin, and 4 μm sections were cut for staining. For morphometry, infarct regions were identified by Picrosirius red staining; human graft was identified by anti-human cardiac troponin T (Invitrogen, MA5-12960), stained using avidin-biotin reaction (ABC Kit, VectorLabs), followed by chromogenic detection via diaminobenzidine (Sigmafast, Sigma Life Science) (Figure S3). The slides were digitized using a whole slide scanner (Nanozoomer, Hamamatsu), and the images were viewed and exported with NDP.view 2.6.13 (Hamamatsu). Areas of infarct and graft were analyzed using a custom-written algorithm in the ImageJ open source software platform (Schindelin et al., 2015). In brief, after extracting images in TIFF format (Deroulers et al., 2013), the image foreground was segmented by a threshold derived from the distribution in brightness of its pixels, resulting in a binary mask that delineates the imaged tissue section. Subsequent color de-convolution by thresholding hue, brightness, and saturation allowed segmentation of regions stained by Picrosirius red stain or areas immunolabeled for human cardiac troponin T. To separate scar from diffuse fibrosis, a cutoff for particle size was applied. Infarct size and graft size were calculated (percent area × block weight), summed for the entire ventricle, and expressed as a percentage of left ventricular mass or infarct mass, respectively. Please see the supplemental information for Purkinje fiber staining.

## Author contributions

K.N. conceived the study, led experimental design, performed surgical and percutaneous procedures, analyzed and interpreted data, and wrote the manuscript. L.E.N. assisted with the conception, experimental design, and execution of the study, performed surgical and percutaneous procedures, supervised veterinary care, contributed to data analysis, and edited the manuscript. X.Y. performed histologic analysis and edited the manuscript. G.J.W. developed the experimental procedures, performed histologic analysis, and contributed to the manuscript. D.E.-N. performed histologic analysis and contributed to the manuscript. H.T. assisted with surgical and percutaneous procedures, assisted with experimental design, and performed histologic analysis. S.D. assisted with surgical and percutaneous procedures and performed histologic analysis. F.A.K. cultured and characterized the hESC-CM cell product. A.J. cultured and characterized the hESC-CM cell product. A.F.-T. cultured and characterized the hESC-CM cell product. D.S.N. assisted with experimental design and writing the manuscript. S.M. assisted with experimental design and edited the manuscript. A.B. assisted with experimental design and edited the manuscript. M.R.R. assisted with the study's conception, experimental design, and execution. K.C. performed statistical analysis and contributed to the manuscript. R.T. supervised histologic analysis and development of experimental procedure. H.R. assisted with the study's conception, experimental design, and execution. L.P. assisted with the study's conception, experimental design, and execution. B.C.K. assisted with the study's conception and experimental design. S.K. with assisted the study's experimental design and execution and supervised cell manufacturing. R.S.T. assisted with the study's conception, experimental design, and execution, supervised cell manufacturing, and obtained research funding. W.R.M. assisted with the study's conception, experimental design, and execution, and obtained research funding. C.E.M. conceived and supervised the study, obtained research funding, and contributed to data analysis and writing of the manuscript.

## Conflicts of interest

K.N., M.R.R., B.C.K., and W.R.M. are advisors to Sana Biotechnology. D.E.-N., H.T., S.D., F.A.K., A.J., A.F.-T., D.S.N., S.K., R.S.T., and C.E.M. are employees of Sana Biotechnology. C.E.M. is a scientific founder and equity holder of Sana Biotechnology. The remaining authors have nothing to disclose. These studies were supported by the UW Medicine Heart Regeneration Program, the 10.13039/100001906Washington Research Foundation, and a gift from Mike and Lynn Garvey (all Seattle, WA). This work also was supported in part by 10.13039/100000002NIH grants R01HL128362, and a grant from the Fondation Leducq Transatlantic Network of Excellence (Boston, MA; to C.E.M.) and the Bruce-Laughlin Research Fellowship (Seattle, WA; to K.N.).

## Supplemental information

Document S1. Supplemental experimental procedures, Figures S1–S4, and Table S1

Document S2. Article plus supplemental information

## Footnotes

Supplemental information can be found online at https://doi.org/10.1016/j.stemcr.2021.08.005.

## References

[bib1] Bedada F.B., Chan S.S., Metzger S.K., Zhang L., Zhang J., Garry D.J., Kamp T.J., Kyba M., Metzger J.M. (2014). Acquisition of a quantitative, stoichiometrically conserved ratiometric marker of maturation status in stem cell-derived cardiac myocytes. Stem Cell Reports.

[bib2] Caspi O., Huber I., Kehat I., Habib M., Arbel G., Gepstein A., Yankelson L., Aronson D., Beyar R., Gepstein L. (2007). Transplantation of human embryonic stem cell-derived cardiomyocytes improves myocardial performance in infarcted rat hearts. J. Am. Coll. Cardiol..

[bib3] Chen V.C., Ye J., Shukla P., Hua G., Chen D., Lin Z., Liu J.C., Chai J., Gold J., Wu J. (2015). Development of a scalable suspension culture for cardiac differentiation from human pluripotent stem cells. Stem Cell Res.

[bib4] Chong J.J., Yang X., Don C.W., Minami E., Liu Y.W., Weyers J.J., Mahoney W.M., Van Biber B., Cook S.M., Palpant N.J. (2014). Human embryonic-stem-cell-derived cardiomyocytes regenerate non-human primate hearts. Nature.

[bib5] Chow E., Woodard J.C., Farrar D.J. (1990). Rapid ventricular pacing in pigs: an experimental model of congestive heart failure. Am. J. Physiol..

[bib6] Deroulers C., Ameisen D., Badoual M., Gerin C., Granier A., Lartaud M. (2013). Analyzing huge pathology images with open source software. Diagn. Pathol..

[bib7] Eschenhagen T., Bolli R., Braun T., Field L.J., Fleischmann B.K., Frisen J., Giacca M., Hare J.M., Houser S., Lee R.T. (2017). Cardiomyocyte regeneration: a consensus statement. Circulation.

[bib8] Garcia-Bustos V., Sebastian R., Izquierdo M., Molina P., Chorro F.J., Ruiz-Sauri A. (2017). A quantitative structural and morphometric analysis of the Purkinje network and the Purkinje-myocardial junctions in pig hearts. J. Anat..

[bib9] Guo Y., Pu W.T. (2020). Cardiomyocyte maturation: new phase in development. Circ. Res..

[bib10] Ichimura H., Kadota S., Kashihara T., Yamada M., Ito K., Kobayashi H., Tanaka Y., Shiba N., Chuma S., Tohyama S. (2020). Increased predominance of the matured ventricular subtype in embryonic stem cell-derived cardiomyocytes in vivo. Sci. Rep..

[bib11] Josephson M., Marchlinski F., Buxton A., Waxman H., Doherty J., Kienzle M., Falcone R. (1984). Tachycardias: Mechanisms, Diagnosis, Treatment.

[bib12] Kadota S., Pabon L., Reinecke H., Murry C.E. (2017). In vivo maturation of human induced pluripotent stem cell-derived cardiomyocytes in neonatal and adult rat hearts. Stem Cell Reports.

[bib13] Kannan S., Kwon C. (2020). Regulation of cardiomyocyte maturation during critical perinatal window. J. Physiol..

[bib14] Karbassi E., Fenix A., Marchiano S., Muraoka N., Nakamura K., Yang X., Murry C.E. (2020). Cardiomyocyte maturation: advances in knowledge and implications for regenerative medicine. Nat. Rev. Cardiol..

[bib15] Laflamme M.A., Chen K.Y., Naumova A.V., Muskheli V., Fugate J.A., Dupras S.K., Reinecke H., Xu C., Hassanipour M., Police S. (2007). Cardiomyocytes derived from human embryonic stem cells in pro-survival factors enhance function of infarcted rat hearts. Nat. Biotechnol..

[bib16] Lelovas P.P., Kostomitsopoulos N.G., Xanthos T.T. (2014). A comparative anatomic and physiologic overview of the porcine heart. J. Am. Assoc. Lab Anim. Sci..

[bib17] Liu Y.W., Chen B., Yang X., Fugate J.A., Kalucki F.A., Futakuchi-Tsuchida A., Couture L., Vogel K.W., Astley C.A., Baldessari A. (2018). Human embryonic stem cell-derived cardiomyocytes restore function in infarcted hearts of non-human primates. Nat. Biotechnol..

[bib18] Marchiano S., Bertero A., Murry C.E. (2019). Learn from your elders: developmental biology lessons to guide maturation of stem cell-derived cardiomyocytes. Pediatr. Cardiol..

[bib19] Maroli G., Braun T. (2020). The long and winding road of cardiomyocyte maturation. Cardiovasc. Res..

[bib20] Mostow N.D., Rakita L., Vrobel T.R., Noon D.L., Blumer J. (1984). Amiodarone: correlation of serum concentration with suppression of complex ventricular ectopic activity. Am. J. Cardiol..

[bib21] Nakamura K., Murry C.E. (2019). Function follows form—a review of cardiac cell therapy. Circ. J..

[bib22] Pallante B.A., Giovannone S., Fang-Yu L., Zhang J., Liu N., Kang G., Dun W., Boyden P.A., Fishman G.I. (2010). Contactin-2 expression in the cardiac Purkinje fiber network. Circ. Arrhythm Electrophysiol..

[bib23] Panescu D., Kroll M., Brave M. (2014). Limitations of animal electrical cardiac safety models. Annu. Int. Conf. IEEE Eng. Med. Biol. Soc..

[bib24] Romagnuolo R., Masoudpour H., Porta-Sanchez A., Qiang B., Barry J., Laskary A., Qi X., Masse S., Magtibay K., Kawajiri H. (2019). Human embryonic stem cell-derived cardiomyocytes regenerate the infarcted pig heart but induce ventricular tachyarrhythmias. Stem Cell Reports.

[bib25] Schindelin J., Rueden C.T., Hiner M.C., Eliceiri K.W. (2015). The ImageJ ecosystem: an open platform for biomedical image analysis. Mol. Reprod. Dev..

[bib26] Shiba Y., Fernandes S., Zhu W.Z., Filice D., Muskheli V., Kim J., Palpant N.J., Gantz J., Moyes K.W., Reinecke H. (2012). Human ES-cell-derived cardiomyocytes electrically couple and suppress arrhythmias in injured hearts. Nature.

[bib27] Shiba Y., Filice D., Fernandes S., Minami E., Dupras S.K., Biber B.V., Trinh P., Hirota Y., Gold J.D., Viswanathan M., Laflamme M.A. (2014). Electrical integration of human embryonic stem cell-derived cardiomyocytes in a guinea pig chronic infarct model. J. Cardiovasc. Pharmacol. Ther..

[bib28] Shiba Y., Gomibuchi T., Seto T., Wada Y., Ichimura H., Tanaka Y., Ogasawara T., Okada K., Shiba N., Sakamoto K. (2016). Allogeneic transplantation of iPS cell-derived cardiomyocytes regenerates primate hearts. Nature.

[bib29] Staubli M., Bircher J., Galeazzi R.L., Remund H., Studer H. (1983). Serum concentrations of amiodarone during long term therapy. Relation to dose, efficacy and toxicity. Eur. J. Clin. Pharmacol..

[bib30] van der Spoel T.I., Jansen of Lorkeers S.J., Agostoni P., van Belle E., Gyongyosi M., Sluijter J.P., Cramer M.J., Doevendans P.A., Chamuleau S.A. (2011). Human relevance of pre-clinical studies in stem cell therapy: systematic review and meta-analysis of large animal models of ischaemic heart disease. Cardiovasc. Res..

[bib31] van Laake L.W., Passier R., Monshouwer-Kloots J., Verkleij A.J., Lips D.J., Freund C., den Ouden K., Ward-van Oostwaard D., Korving J., Tertoolen L.G. (2007). Human embryonic stem cell-derived cardiomyocytes survive and mature in the mouse heart and transiently improve function after myocardial infarction. Stem Cell Res..

[bib32] Velagaleti R.S., Pencina M.J., Murabito J.M., Wang T.J., Parikh N.I., D'Agostino R.B., Levy D., Kannel W.B., Vasan R.S. (2008). Long-term trends in the incidence of heart failure after myocardial infarction. Circulation.

[bib33] Waks J.W., Zimetbaum P. (2017). Antiarrhythmic drug therapy for rhythm control in atrial fibrillation. J. Cardiovasc. Pharmacol. Ther..

[bib34] Yu J.K., Franceschi W., Huang Q., Pashakhanloo F., Boyle P.M., Trayanova N.A. (2019). A comprehensive, multiscale framework for evaluation of arrhythmias arising from cell therapy in the whole post-myocardial infarcted heart. Sci. Rep..

