# Supplementary material for: Pharmacologic therapy for engraftment arrhythmia induced by transplantation of human cardiomyocytes
Source: Stem Cell Reports. 2021 Sep 9;16(10):2473–87. doi: 10.1016/j.stemcr.2021.08.005 (PMC8514851; doi:10.1016/j.stemcr.2021.08.005)
Supplement: Document S1. Supplemental experimental procedures, Figures S1–S4, and Table S1 [file mmc1.pdf]

Stem Cell Reports, Volume *16*

Supplemental Information

Pharmacologic therapy for engraftment arrhythmia induced by transplantation of human cardiomyocytes

Kenta Nakamura, Lauren E. Neidig, Xiulan Yang, Gerhard J. Weber, Danny El-Nachef, Hiroshi Tsuchida, Sarah Dupras, Faith A. Kalucki, Anu Jayabalu, Akiko Futakuchi-Tsuchida, Daisy S. Nakamura, Silvia Marchianò, Alessandro Bertero, Melissa R. Robinson, Kevin Cain, Dale Whittington, Rong Tian, Hans Reinecke, Lil Pabon, Björn C. Knollmann, Steven Kattman, R. Scott Thies, W. Robb MacLellan, and Charles E. Murry

Supplemental Figures

Figure S1

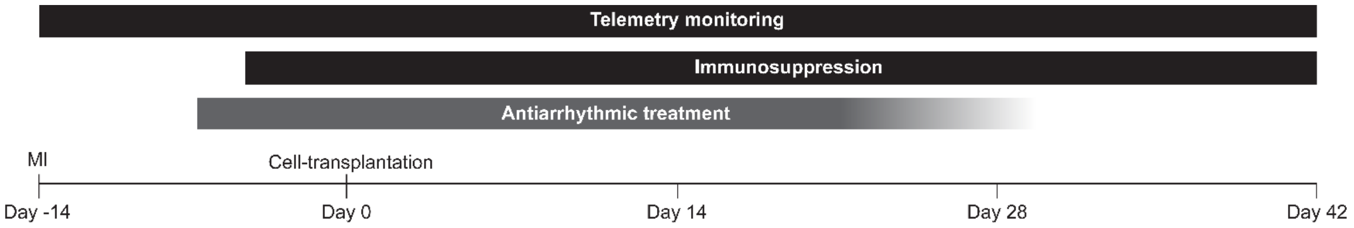

**Immunosuppression**	
Calcineurin inhibitor	Cyclosporine PO (trough goal >300 ng/mL)
Glucocorticoid	Methylprednisolone 1.5 mg/kg PO QD x2 week, 1 mg/kg thereafter
CTLA4 inhibitor	CTLA4-Ig (abatacept) 12.5 mg/kg IV q 2 weeks
Prophylaxis	Cephalexin, sulfamethoxazole/trimethoprim, ganciclovir, probiotics

**Antiarrhythmic treatment**	
Rhythm/rate control	Amiodarone 200–1200 mg BID PO (steady state serum goal 1.5–2.5 μg/mL)
Rate control	Ivabradine 2.5–15 mg PO BID for HR ≥150 bpm (goal <125 bpm)

Study timeline for Phase 2 drug trial of chronic amiodarone and adjunctive ivabradine therapy. Myocardial infarction (MI) was induced by 90-minute balloon occlusion of the mid-left anterior descending artery two weeks prior to human embryonic stem cell-derived cardiomyocyte transplantation (day 0). All subjects received multi-drug immunosuppression. Treated cohort received rate and rhythm control with combined oral amiodarone and adjunctive oral ivabradine.

Figure S2

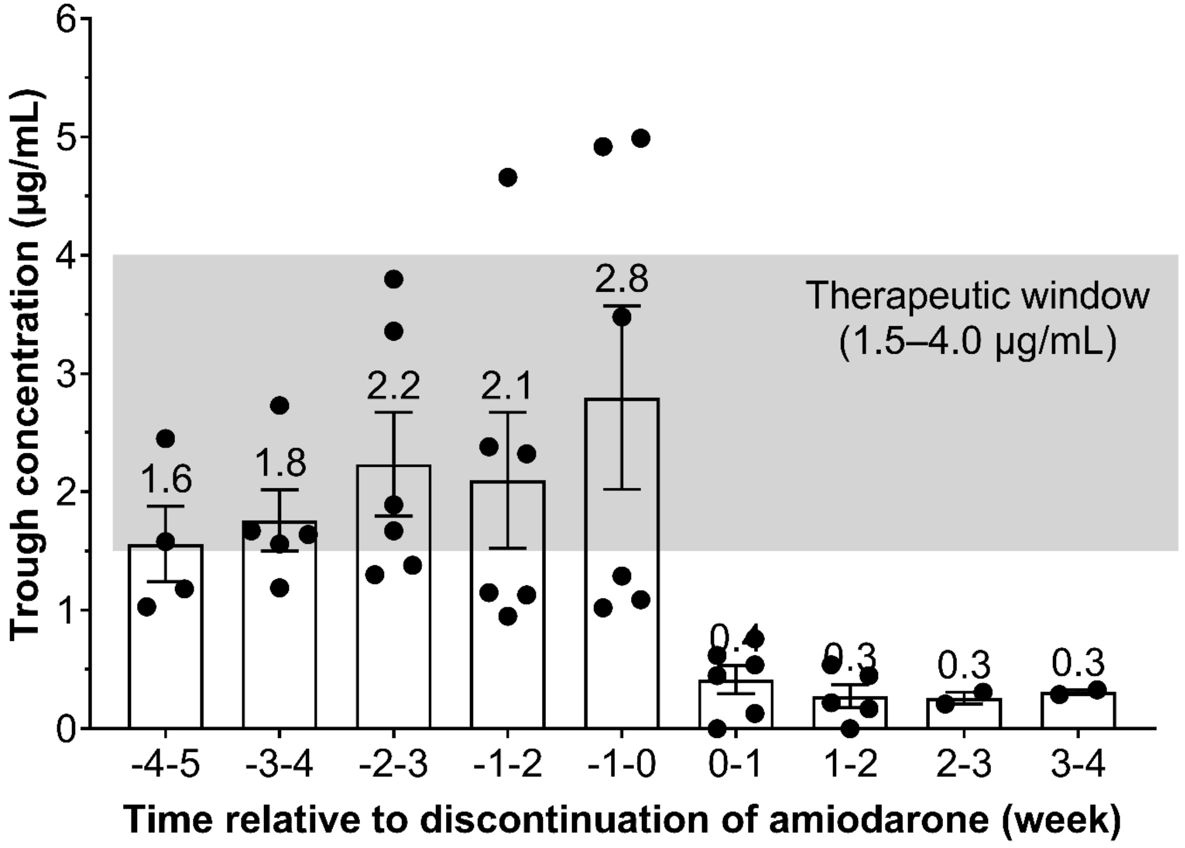

Plasma amiodarone levels in pigs. Amiodarone levels were measured in plasma by a custom liquid chromatography-mass spectrometry assay. Chronic oral amiodarone in six pigs was discontinued after achieving electrical maturation and stabilization of engraftment arrythmia. Serum through concentrations of amiodarone were assayed weekly including 3–4 weeks after discontinuation.

Figure S3

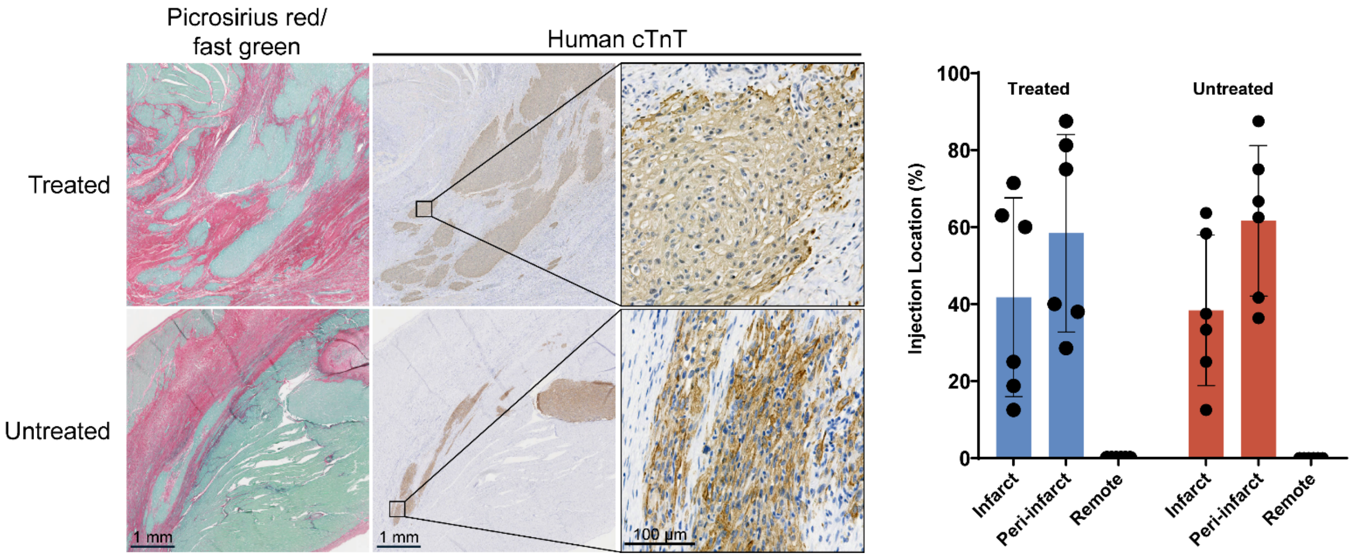

hESC-CM graft histology and location. Left panel: Histological sections stained with picrosirius red to identify collagen (infarct) and fast green to identify viable myocardium. Adjacent sections labeled with human cTnT (brown) identify transplanted hESC-CM graft within unstained porcine myocardium and scar tissue. Sections from both treated and untreated subjects were obtained on post-transplantation day 42. Right panel: Transplanted hESC-CM grafts were located similarly between treated (blue) and untreated (red) cohorts and successfully targeted the infarct and peri-infarct regions of the anterior wall.

Figure S4

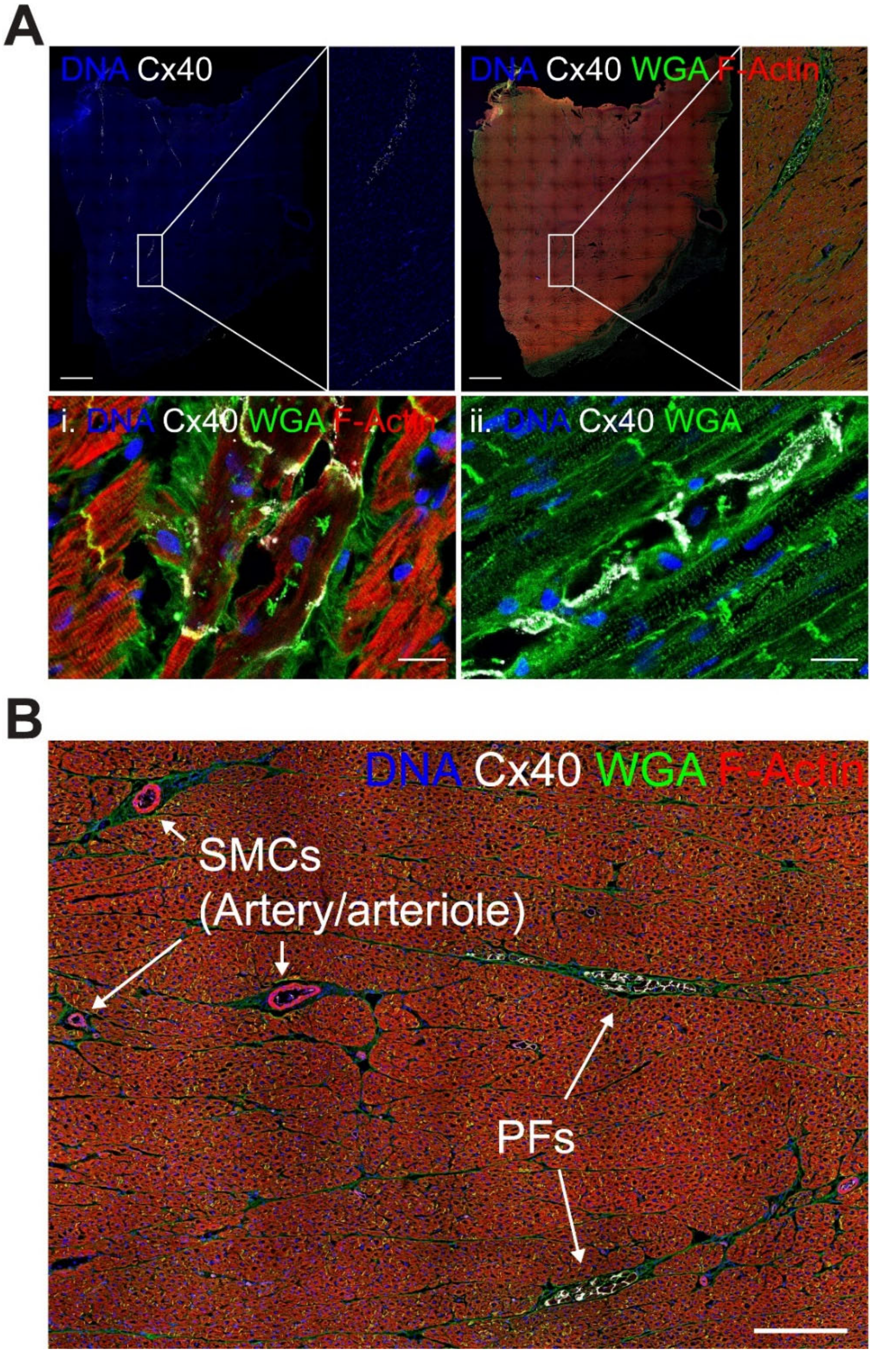

Purkinje fibers are distributed in a mesh-like network throughout the native porcine myocardium and are specifically marked by Connexin 40. Subendocardial and intramyocardial connexin 40 (Cx40)-positive Purkinje fibers (PFs, white) in transverse section of left ventricular free wall, scale bar 2 mm (A). Intramyocardial PFs are shown with higher magnification insets. Further magnified view of white boxed regions show Cx40 localizes to gap junctions of Purkinje cells that display lower sarcomere content (F-Actin, red) (i.) and lack T-Tubules (WGA, green) (ii.) in contrast to surrounding cardiomyocytes, scale bar 20 µm. Cx40 specifically marks myocardial Purkinje fibers (PFs) diffusely distributed in the anterior left ventricular wall cross section, scale bar 2 mm (B).

Supplemental Table 1

**Lidocaine (Ib)**	100 mg IV	Modest HR effect, rare cardioversion
**Flecainide (Ic)**	2 mg/kg PO, 4 mg/kg PO, 6 mg/kg PO, 10 mg/kg PO	No response on HR or EA burden
**Propafenone (Ic)**	1 mg/kg IV, 2 mg/kg IV, 3 mg/kg IV	No response on HR, transient cardioversion*
**Amiodarone (III)**	150 mg IV	Modest HR effect, frequent cardioversion
**Sotalol (III)**	1 mg/kg PO, 2 mg/kg PO, 4 mg/kg PO	No response on HR or EA burden
**Metoprolol (β_1AR)**	5 mg IV, 25 mg PO BID, 50 mg PO BID, 75 mg PO BID	Moderate HR effect (IV only), no response on EA burden
**Ivabradine (I_f)**	2.5 mg PO, 5 mg PO BID, 10 mg PO BID, 15 mg BID; 1 mg/kg IV, 2 mg/kg IV	Robust dose-dependent HR effect (PO only)**, no response on EA burden

* Severe nausea/emesis observed at therapeutic doses, limiting clinical utility

** Severe bradycardia

Abbreviations: β_1AR, β1-adrenergic receptor; BID, twice daily; HR, heart rate; EA, engraftment arrythmia; I_f, funny current; PO, oral; VT, ventricular tachycardia

Supplemental Experimental Procedures

Animal subject care

All protocols were approved and conducted in accordance with the University of Washington (UW) Office of Animal Welfare and the Institutional Animal Care and Use Committee. Animals received ad libitum water and were fed twice a day (Lab Diet-5084 Laboratory Porcine Grower Diet). For surgical procedures, anesthesia was induced with a combination of intramuscular butorphanol, acepromazine and ketamine. Animals were intubated and mechanically ventilated using isoflurane and oxygen to maintain a surgical plane of anesthesia. Vital signs were monitored continuously throughout each procedure. All animals received subcutaneous Buprenorphine SR-Lab (ZooPharm) for post-operative analgesia and were euthanized by intravenous Euthasol (Virbac). All post-mortem examinations were performed by a blinded board-certified veterinary pathologist.

Porcine myocardial infarction model

Percutaneous ischemia/reperfusion injury was induced as previously described in NHP [1] with modification for the porcine model. A 5–8 cm incision was made in the femoral triangle and the femoral artery was exposed by blunt dissection. Prior to obtaining vascular access, heparin was administered to achieve therapeutic anticoagulation (activated coagulation time > 250 sec). A 5-French guidewire/introducer sheath system (Terumo Medical) was placed into the femoral artery and secured. Continuous ECG, invasive arterial blood pressure, pulse-oximetry and capnography were monitored throughout the procedure. Intravenous amiodarone 150 mg and lidocaine 100 mg were administered as single boluses prior to ischemia to minimize the risk of arrhythmia. Under fluoroscopic guidance (OEC 9800 Plus, GE Medical Systems), a 5-French Judkins right 2 or hockey stick guide catheter (Boston Scientific) was advanced into the ascending aorta to selectively engage the ostium of the left main coronary artery. Coronary angiography was performed using hand injections of contrast (Visipaque) and a 0.014” coronary guidewire (Runthrough NS Extra Floppy, Terumo Medical) was placed into the distal left anterior descending coronary artery (LAD). An appropriately sized angioplasty balloon catheter was then positioned into the mid-LAD distal to the first diagonal branch artery and inflated to the minimum pressure required for total obstruction of distal perfusion as confirmed by angiography. Ischemia was confirmed by ST-segment elevation on the ECG. Animals were maintained under anesthesia with ventilatory and hemodynamic support for 90 minutes, after which the balloon was deflated to restore distal perfusion, again confirmed by fluoroscopy and ECG. The animal was observed for reperfusion arrhythmias and externally cardioverted if ventricular fibrillation occurred. Prior to recovery, all animals received implantable telemetry units and central venous catheter placement. Briefly, the external jugular vein in the jugular furrow was exposed and a 5-French central venous catheter (Access Technologies) was inserted and tunneled out to the dorsal prescapular area. The telemetry transmitter (EMKA easyTEL+) was implanted in a subcutaneous pocket using the same incision in the jugular furrow, and subcutaneous leads were tunneled to capture the cardiac apex to base. The overall procedural mortality including the infarct was < 10%.

Phase 1 study design

Five subjects were administered serial trials of antiarrhythmics (Supplemental Table 1) and observed for acute response by continuous ECG monitoring with the following dosing schedule:

**Subject A**	Ivabradine	Sotalol	Flecainide	Metoprolol	Amiodarone
**Subject B**	Sotalol	Metoprolol	Flecainide	Ivabradine	Lidocaine

**Subject C**	Lidocaine	Ivabradine	Amiodarone		
**Subject D**	Propafenone	Amiodarone			
**Subject E**	Propafenone	Amiodarone			

Antiarrhythmics were trialed after at least two continuous days of EA and continued until the subject became clinical unstable. Dose escalation of the same intravenous agents were delivered as a bolus dose over two minutes with a minimum of 10 minutes between doses through an indwelling central venous catheter. Oral agents were administrated by direct observation in a minimum of apples, apple sauce or pumpkin puree with daily feeding and titrated daily for dose escalation. A washout period of at least three days was provided between agents. Amiodarone was administered as the last agent for testing given concern for prolonged half-life and elimination kinetics. Cardioversion was observed with amiodarone and propafenone, typically immediately or within minutes of intravenous dose administration.

Purkinje fiber histology

For thin sections, tissue was cut and trimmed to 1 cm × 1 cm × 3 mm, snap frozen in isopentane, and embedded in OCT (TissueTek). 10 µm sections were immersed in 100% methanol at -20°C for 15 minutes and stained with standard immunofluorescence technique using stains described below. Images were acquired on a Leica SP8 confocal microscope.
For thick sections, 1 cm × 1 cm × 3 mm pieces of tissue containing graft were incubated in 100% methanol at 20°C for 1 hour, rehydrated (80% methanol, 60% methanol, 0% methanol, diluted in PBS, 15-minute incubation at -20°C for each reagent). 150 µm sections were cut on a Leica VT1200s vibratome and stained with standard immunofluorescence technique using stains described below. Stained sections were then cleared using BABB as previously reported (El-Nachef et al., 2018), and imaged on a Leica SP8 confocal microscope with 1 µm z-step increments.

Purkinje fiber staining

Sections were stained the following reagents: Hoechst 33342 (DNA, Thermo Fisher Scientific, #62249), Wheat germ agglutinin-Oregon Green (WGA, Thermo Fisher Scientific, #W6748), Phalloidin-647 (F-Actin, Thermo Fisher Scientific, #A22287), anti-Connexin 40 (Cx40, Alpha Diagnostics, #CXN40A), or anti-slow skeletal troponin I (ss-TnI, Novus, #NBP2-46170) with one of two anti-rabbit secondary antibodies (Alexa Fluor 555/647, Thermo Fisher Scientific, #A-31570/A-31573).

References

1. Liu YW, Chen B, Yang X, Fugate JA, Kalucki FA, Futakuchi-Tsuchida A, Couture L, Vogel KW, Astley CA, Baldessari A, Ogle J, Don CW, Steinberg ZL, Seslar SP, Tuck SA, Tsuchida H, Naumova AV, Dupras SK, Lyu MS, Lee J, Hailey DW, Reinecke H, Pabon L, Fryer BH, MacLellan WR, Thies RS and Murry CE. Human embryonic stem cell-derived cardiomyocytes restore function in infarcted hearts of non-human primates. *Nat Biotechnol*. 2018;36:597-605.
2. Staubli M, Bircher J, Galeazzi RL, Remund H and Studer H. Serum concentrations of amiodarone during long term therapy. Relation to dose, efficacy and toxicity. *Eur J Clin Pharmacol*. 1983;24:485-94.
3. Mostow ND, Rakita L, Vrobel TR, Noon DL and Blumer J. Amiodarone: correlation of serum concentration with suppression of complex ventricular ectopic activity. *Am J Cardiol*. 1984;54:569-74.
4. Chong JJ, Yang X, Don CW, Minami E, Liu YW, Weyers JJ, Mahoney WM, Van Biber B, Cook SM, Palpant NJ, Gantz JA, Fugate JA, Muskheli V, Gough GM, Vogel KW, Astley CA, Hotchkiss CE, Baldessari A, Pabon L, Reinecke H, Gill EA, Nelson V, Kiem HP, Laflamme MA and Murry CE. Human embryonic-stem-cell-derived cardiomyocytes regenerate non-human primate hearts. *Nature*. 2014;510:273-7.
5. Schindelin J, Rueden CT, Hiner MC and Eliceiri KW. The ImageJ ecosystem: An open platform for biomedical image analysis. *Mol Reprod Dev*. 2015;82:518-29.
6. Deroulers C, Ameisen D, Badoual M, Gerin C, Granier A and Lartaud M. Analyzing huge pathology images with open source software. *Diagn Pathol*. 2013;8:92.
7. El-Nachef D, Oyama K, Wu YY, Freeman M, Zhang Y and MacLellan WR. Repressive histone methylation regulates cardiac myocyte cell cycle exit. *J Mol Cell Cardiol*. 2018;121:1-12.
