# Supplementary material for: Pharmacologic therapy for engraftment arrhythmia induced by transplantation of human cardiomyocytes
Source: Stem Cell Reports. 2021 Sep 9;16(10):2473–87. doi: 10.1016/j.stemcr.2021.08.005 (PMC8514851; doi:10.1016/j.stemcr.2021.08.005)
Supplement: Document S2. Article plus supplemental information [file mmc4.pdf]

Stem Cell Reports
Article
OPEN ACCESS

Pharmacologic therapy for engraftment arrhythmia induced by transplantation of human cardiomyocytes

Kenta Nakamura,[1,2,3] Lauren E. Neidig,[1,2,4] Xiulan Yang,[1,2,5] Gerhard J. Weber,[1,2,3] Danny El-Nachef,[6] Hiroshi Tsuchida,[1,2,5,6] Sarah Dupras,[1,2,5,6] Faith A. Kalucki,[1,2,5,6] Anu Jayabalu,[1,2,5,6] Akiko Futakuchi-Tsuchida,[1,2,5,6] Daisy S. Nakamura,[1,2,3,6] Silvia Marchianò,[1,2,5] Alessandro Bertero,[1,2,5] Melissa R. Robinson,[3] Kevin Cain,[7] Dale Whittington,[8] Rong Tian,[1,9,10] Hans Reinecke,[1,2,5] Lil Pabon,[1,2,5,6] Björn C. Knollmann,[11] Steven Kattman,[1,2,5,6] R. Scott Thies,[1,2,5,6] W. Robb MacLellan,[1,2,3,10] and Charles E. Murry[1,2,3,5,6,10,*]

[1]Institute for Stem Cell and Regenerative Medicine, University of Washington, 850 Republican Street, Brotman Building Room 453, Seattle, WA 98109, USA
[2]Center for Cardiovascular Biology, University of Washington, Seattle, WA 98109, USA
[3]Division of Cardiology, Department of Medicine, University of Washington, Seattle, WA 98195, USA
[4]Department of Comparative Medicine, University of Washington, Seattle, WA 98195, USA
[5]Department of Laboratory Medicine & Pathology, University of Washington, Seattle, WA 98195, USA
[6]Sana Biotechnology, Seattle, WA 98102, USA
[7]Department of Biostatics, University of Washington, Seattle, WA 98195, USA
[8]Department of Medicinal Chemistry, University of Washington, Seattle, WA 98195, USA
[9]Department of Anesthesiology & Pain Medicine, University of Washington, Seattle, WA 98195, USA
[10]Department of Bioengineering, University of Washington, Seattle, WA 98195, USA
[11]Division of Clinical Pharmacology, Vanderbilt University School of Medicine, Nashville, TN 37232, USA
*Correspondence: murry@uw.edu
https://doi.org/10.1016/j.stemcr.2021.08.005

SUMMARY

Heart failure remains a significant cause of morbidity and mortality following myocardial infarction. Cardiac remuscularization with transplantation of human pluripotent stem cell-derived cardiomyocytes is a promising preclinical therapy to restore function. Recent large animal data, however, have revealed a significant risk of engraftment arrhythmia (EA). Although transient, the risk posed by EA presents a barrier to clinical translation. We hypothesized that clinically approved antiarrhythmic drugs can prevent EA-related mortality as well as suppress tachycardia and arrhythmia burden. This study uses a porcine model to provide proof-of-concept evidence that a combination of amiodarone and ivabradine can effectively suppress EA. None of the nine treated subjects experienced the primary endpoint of cardiac death, unstable EA, or heart failure compared with five out of eight (62.5%) in the control cohort (hazard ratio = 0.00; 95% confidence interval: 0–0.297; p = 0.002). Pharmacologic treatment of EA may be a viable strategy to improve safety and allow further clinical development of cardiac remuscularization therapy.

INTRODUCTION

Ischemic heart disease, including myocardial infarction (MI) and heart failure, remains the leading cause of death in the United States and around the world. Approximately one billion cardiomyocytes are permanently lost during MI and an increasing proportion of MI survivors—an estimated 20% to 30% (Velagaleti et al., 2008)—later develop heart failure. Current treatments can slow the initiation and progression of heart failure, but none replaces lost myocardium, short of orthotopic heart transplantation, which remains restricted in availability and indication (Nakamura and Murry, 2019). Human pluripotent stem cells ([hPSCs], comprising embryonic stem cells [ESCs] and their reprogrammed cousins, induced pluripotent stem cells [iPSCs]) are a renewable source of cardiomyocytes (CMs). Transplantation of hPSC-derived cardiomyocytes (hPSC-CMs) into infarcted myocardium of small animals—mice, rats, and guinea pigs—has shown stable engraftment (Caspi et al., 2007; Laflamme et al., 2007; Shiba et al., 2012, 2014; van Laake et al., 2007). More recently, our group and others have shown remuscularization and functional benefit in infarcted non-human primates (NHPs) following transplantation of pluripotent stem cell-derived cardiomyocytes (Chong et al., 2014; Liu et al., 2018; Shiba et al., 2016). In addition to functional remuscularization, the human graft vascularizes and electromechanically couples with the host myocardium within 1 month post-transplant and remains durable up to 3 months, the longest time tested.

Although no arrhythmias were observed in smaller animals, we and others consistently observe ventricular arrhythmias following hPSC-CM transplantation in NHPs (Chong et al., 2014; Liu et al., 2018; Shiba et al., 2016) and pigs (Romagnuolo et al., 2019), which we have called "engraftment arrhythmias" (EAs). EAs are generally transient, occurring within a week of transplantation and typically resolve spontaneously after approximately 1 month. Based on electrical mapping, overdrive pacing, and cardioversion studies, EAs appear to originate focally in the graft

This is an open access article under the CC BY license (http://creativecommons.org/licenses/by/4.0/).

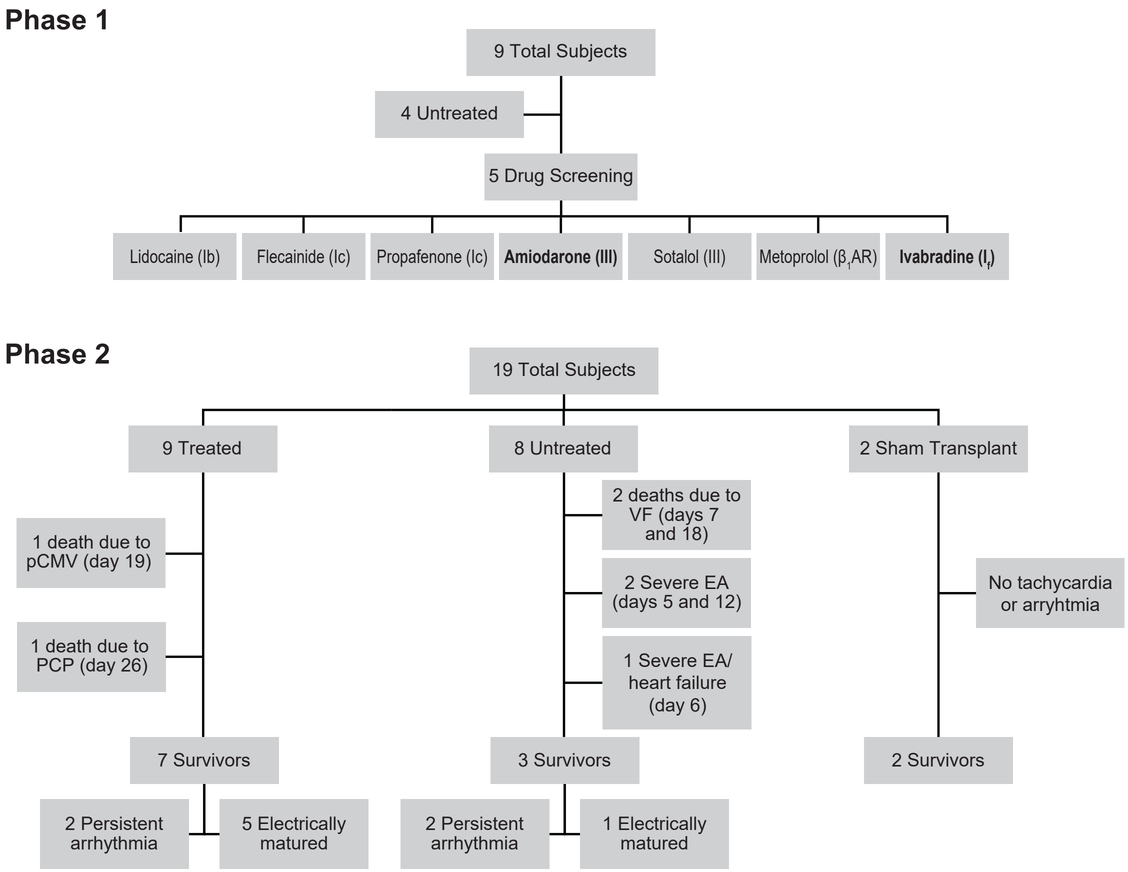

Figure 1. Flowchart of the study design

Phase 1 consisted of nine total subjects, four untreated with any antiarrhythmic to study the natural history of EA and five used to screen seven candidate antiarrhythmic agents. Amiodarone and ivabradine were found to have promising signals of effect and advanced for further study. Phase 2 consisted of 19 total subjects: 9 treated with amiodarone and ivabradine and 8 untreated following hESC-CM transplantation, and 2 untreated following sham transplantation.

or peri-graft myocardium and function as automatous foci rather than reentrant pathways (Liu et al., 2018; Romagnuolo et al., 2019). Although EA is reasonably well tolerated in NHPs, the Laflamme group (Romagnuolo et al., 2019) reported that EA can be lethal in pigs. For this reason, EA has emerged as the biggest impediment to the clinical translation of human cardiomyocyte transplantation (Eschenhagen et al., 2017).

We hypothesized that the risk of EA may be mitigated by treatment with clinically available antiarrhythmic drugs. Because the pig shows heightened sensitivity to EAs, is a well-established model in cardiovascular research (Lelovas et al., 2014) and cell therapy (van der Spoel et al., 2011), whose larger heart permits the use of percutaneous delivery catheters, we chose to test the hypothesis in this large animal model. In the first phase of our study, we screened a panel of seven antiarrhythmic agents. The broad-acting (class III) antiarrhythmic amiodarone and the pacemaker inhibitor (class 0) ivabradine emerged independently as the most promising agents for control of rhythm and rate, respectively. We therefore performed a second phase to test the effect of combined amiodarone and ivabradine treatment. We found that this regimen reduced sudden cardiac death, as well as suppressed tachycardia and arrhythmia.

RESULTS

Clinical history of EA

A flow chart for all subjects in the study is shown in Figure 1 and clinical summaries are provided in Table 1. No significant arrhythmias were noted in the two untreated sham transplant control subjects (9 and 10) that underwent MI and percutaneous intracardiac injection of vehicle. All subjects that received human cardiomyocyte grafts developed EA between 2 and 6 days following cell transplantation. Initiation of EA was characterized by salvos of non-sustained ventricular tachycardia (VT), and this typically

Table 1. Characteristics of study subjects and clinical history

Subject	Age (days)	Weight (kg)	MI	Cell line	Approach	hESC-CM cTnT (%)	hESC-CM viability (%)	Infarct size (%)[a]	Graft size (%)[b]	Graft mass (g)	LV mass (g)	HR (bpm) Day 7	HR (bpm) Day 30	Arrhythmia burden (% of time) Day 7	Arrhythmia burden (% of time) Day 30	Outcome
**Treated**																
3	231	34.7	yes	H7	Surg	98	89	6.8	5.4	0.5	125.9	73	N/A	2.0	N/A	euthanasia, day 26 (PCP)
4	227	32.0	yes	H7	Perc	98	89	8.2	7.3	0.8	137.9	93	94	86.0	97.8	survival
5	236	33.0	yes	RUES2	Perc	91	89	7.1	9.0	0.5	84.0	163	81	25.6	0.5	survival
6	231	33.4	yes	RUES2	Perc	91	90	10.1	1.4	0.1	94.2	100	N/A	47.1	N/A	euthanasia, day 19 (pCMV)
7	230	37.0	yes	RUES2	Perc	91	93	9.6	0.3	0.0	142.7	89	79	45.8	28.2	survival
8	299	33.5	yes	RUES2	Perc	86	88	15.9	3.4	0.8	153.3	73	77	67.6	4.7	survival
15	271	33.0	yes	RUES2	Perc	89	88	10.9	4.2	0.5	113.4	79	76	35.4	44.9	survival
16	263	34.0	yes	RUES2	Perc	88	85	14.6	0.7	0.1	136.7	74	69	43.9	7.6	survival
18	380	32.5	yes	RUES2	Perc	94	75	9.3	3.7	0.5	144.5	69	78	1.6	1.6	survival
Avg ± SEM	263.1 ± 16.8	33.7 ± 0.5				92 ± 1	87 ± 2	10.3 ± 1	3.9 ± 1	0.4 ± 0.1	125.8 ± 8	90 ± 10	79 ± 3	39.5 ± 9.2	26.5 ± 13.4	
**Untreated**																
1	267	32.0	no	H7	Surg	98	88	N/A	N/A	0.3	75.10	328	N/A	100.0	N/A	primary endpoint day 7 (VF)
2	314	32.0	yes	H7	Surg	98	90	5.8	5.6	0.3	92.80	120	N/A	62.7	N/A	primary endpoint day 19 (VF)
11	284	35.0	yes	RUES2	Perc	86	90	13.2	1.0	0.2	134.9	N/A	N/A	N/A	N/A	primary endpoint day 5 (EA)
12	283	33.5	yes	RUES2	Perc	82	87	9.7	1.1	0.1	112.4	166	N/A	94.0	N/A	primary endpoint day 12 (EA/HF)
13	292	35.5	yes	RUES2	Perc	87	83	16.5	1.7	0.3	107.4	162	83	95.9	1.9	survival
14	270	33.0	yes	RUES2	Perc	87	90	4.6	9.1	0.4	93.2	N/A	N/A	N/A	N/A	primary endpoint day 6 (EA)
17	209	33.0	yes	RUES2	Perc	98	70	13.5	0.4	0.1	104.3	106	115	49.1	75.7	survival
19	192	33.5	yes	RUES2	Perc	92	74	5.4	3.6	0.3	139.2	98	84	71.5	51.7	survival

(Continued on next page)

Table 1. Continued

Subject	Age (days)	Weight (kg)	MI	Cell line	Approach	hESC-CM cTnT (%)	hESC-CM viability (%)	Infarct size (%)[a]	Graft size (%)[b]	Graft mass (g)	LV mass (g)	HR (bpm) Day 7	HR (bpm) Day 30	Arrhythmia burden (% of time) Day 7	Arrhythmia burden (% of time) Day 30	Outcome
Avg ± SEM	263.9 ± 14.8	33.4 ± 0.4				91 ± 2	84 ± 3	9.8 ± 1.8	3.2 ± 1.2	0.2 ± 0	107.4 ± 7.6	163 ± 35	94 ± 11	78.9 ± 8.5	43.1 ± 21.7	
p value[c]	0.97	0.73				0.77	0.31	0.82	0.64	0.08	0.12	0.03	0.09	0.01	0.52	
**Sham transplant control**																
9	249	33.5	yes	N/A	Perc	N/A	N/A	–	N/A	N/A	N/A	71	68	0.8	0.0	survival
10	233	33.0	yes	N/A	Perc	N/A	N/A	–	N/A	N/A	N/A	77	69	1.2	0.8	survival
Avg ± SEM	241 ± 8	33.3 ± 0.3										74 ± 3	69 ± 1	1.0 ± 0.2	0.4 ± 0.4	

bpm, beats per minute; CK, cytokeratin; EA, engraftment arrhythmia; hESC-CM, human embryonic stem cell-derived cardiomyocytes; HF, heart failure; HR, heart rate; MI, myocardial infarction; N/A, not applicable; surg, surgery; PCP, *Pneumocystis* pneumonia; pCMV, porcine cytomegalovirus; perc, percutaneous; VF, ventricular fibrillation.
[a]% of left ventricle.
[b]% of infarct area.
[c]Treated versus untreated.

progressed to periods of sustained VT with rates ranging from 110 to 250 bpm (Figure 2). The VT was often polymorphic, with the same subject showing different electrical axes and both wide and narrow complex tachycardia at different times. In four of the eight untreated subjects, EA was either fatal or necessitated euthanasia due to a prespecified endpoint of unstable tachycardia (defined as sustained heart rate >350 bpm). In one additional untreated case (subject 12), acute heart failure was noted clinically after a week of EA at a persistent rate of 300 bpm and, based on recommendations from veterinary staff, the subject was euthanized. Signs of heart failure were subsequently confirmed on necropsy. In all other cases, EA was noted with a rapid acceleration to >350 bpm (subjects 11 and 14) and, in two cases, deterioration to ventricular fibrillation (VF) prior to euthanasia (subjects 1 and 2) (Table 1). Three out of four arrhythmic endpoints occurred within the first 3 days of developing EA, and they occurred when tachyarrhythmia was nearly constant. Mean heart rate peaked at day 8 post-transplantation and thereafter began to decline, whereas the arrhythmia burden plateaued from days 8 to 16 and began to normalize thereafter. Of the three survivors in the untreated cohort, two did not normalize rhythm and experienced, on average, 42% arrhythmia burden at the end of study (subjects 17 and 19). The single subject in the untreated cohort that normalized heart rate and rhythm did so on post-transplant day 25 (subject 13).

Screening drugs for antiarrhythmic effects

In phase 1 of the study, we screened six canonical antiarrhythmic agents broadly targeting sodium channels, potassium channels, and β-adrenergic receptors: lidocaine (class Ib Vaughan-Williams-Singh antiarrhythmic, sodium channel inhibitor), flecainide (Ic, sodium channel inhibitor), propafenone (Ic, sodium channel inhibitor), amiodarone (III, potassium channel inhibitor), sotalol (III, potassium channel inhibitor), and metoprolol (β1-adrenergic receptor inhibitor) for effect on EA heart rate and rhythm. In addition, the funny current/HCN4 channel antagonist, ivabradine, was tested (Table S1). This series was not meant to be comprehensive but rather to rapidly identify candidate agents. Animals were brought into the laboratory while in EA, anesthetized, and the effects of short-term intravenous infusion or oral treatment of antiarrhythmic agents were studied. In three instances, intravenous amiodarone successfully cardioverted unstable EA from >350 bpm to a lower heart rate, typically including brief episodes of sinus rhythm (Figure 3A). Oral ivabradine demonstrated robust dose-dependent effects on heart rate but did not restore sinus rhythm (Figure 3B). Four of the other drugs had no significant effect in this screen (lidocaine, flecainide, sotalol, and metoprolol). Propafenone briefly reduced heart

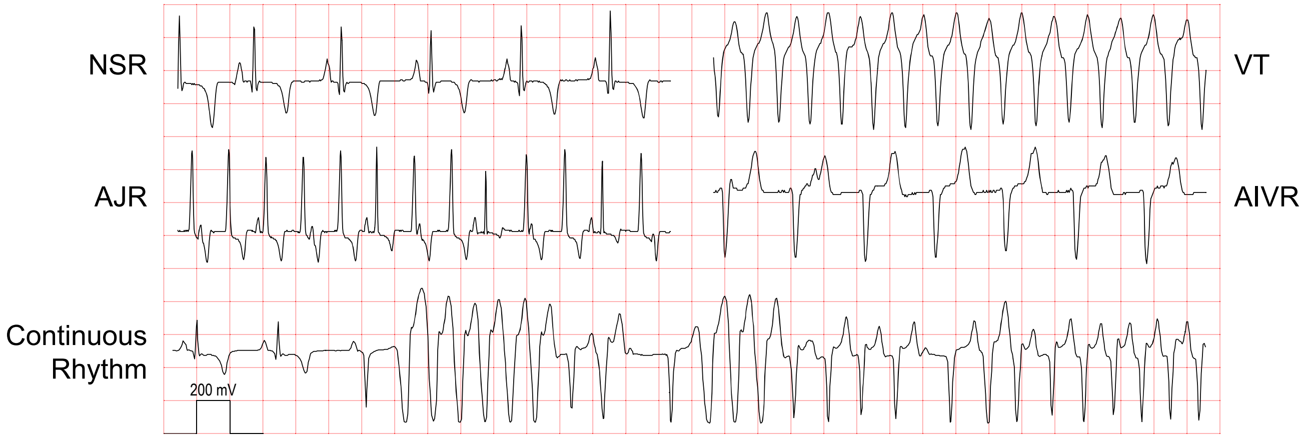

Figure 2. Variable morphologies of EA in a single minipig

Examples of normal sinus rhythm (NSR) and three morphologies of EA resembling accelerated junctional rhythm (AJR), ventricular tachycardia (VT), and accelerated idioventricular rhythm (AIVR) were observed in a single untreated subject (12). Note the variation in rate, electrical axis, and QRS duration. A continuous rhythm recording below exhibits polymorphic EA with QRS complexes varying in rate, duration, and electrical axis. No sustained arrythmias were noted in the sham transplant controls. One box vertical 200 mV, horizontal 0.2 s.

rate and restored sinus rhythm in two-drug challenges, but this drug was associated with substantial gastrointestinal toxicity and not studied further (data not shown).

Amiodarone-ivabradine enhance survival

Given their distinct mechanisms of action and complementary effects on heart rate and rhythm, we formally tested the hypothesis that amiodarone along with ivabradine would reduce a combined primary endpoint of cardiac death, unstable EA >350 bpm, and heart failure in phase 2 of the study. A total of nine treated, eight untreated, and two sham transplant subjects were enrolled in the study with similar baseline and cell transplantation characteristics (Table 1). As detailed in the experimental procedures, treated animals received bolus and maintenance doses of amiodarone, and ivabradine was given as needed to keep heart rates <150 bpm. All treated subjects survived without the primary cardiac endpoint compared with 3/8 (37.5%) of untreated subjects (Figure 4A). The hazard ratio of the primary endpoint was 0.000 (95% CI: 0.000–0.297; $p = 0.002$) with antiarrhythmic treatment. Of note, two of the treated subjects (3 and 6) experienced non-cardiac death at post-transplant days 26 and 19 due to immunosuppression-related complications (*Pneumocystis* pneumonia and porcine cytomegalovirus, respectively). Intention-to-treat analysis of overall survival also favored the treated cohort with hazard ratio of 0.212 (95% CI: 0.030–1.007; $p = 0.05$) (Figure 4B).

Suppression of tachycardia and arrhythmia burden

Pooled and individual subject-level data of heart rate and arrhythmia burden are provided in Figures 5A–5D, respectively. The average heart rate was significantly lower with antiarrhythmic treatment compared with no treatment. Mean heart rates peaked at post-transplantation day 7 in untreated animals at 163 ± 35 bpm, versus average heart rates of 90 ± 10 bpm in the treated cohort ($p = 0.03$) (Table 1; Figure 5A). Heart rate in the treated animals was not significantly different than the normal resting heart rate before MI and transplant (84 ± 1 bpm, $p = 0.21$). Following transplantation, peak daily heart rate for the study duration averaged 305 ± 29 bpm in untreated animals, whereas treatment significantly attenuated peak heart rate to 185 ± 9 bpm ($p = 0.001$) (Figure 5E). We defined arrhythmia burden as the percentage of the day spent in arrhythmia. Treatment reduced peak arrhythmia burden from 96.8% ± 2.9% to 78.1% ± 7.2% ($p = 0.04$), and the average daily burden was reduced from 61.4 ± 7.1% to 27.1 ± 7.0% ($p = 0.004$) (Figure 5F). No differences in heart rate or arrhythmia burden were noted at post-transplant day 30, as the majority of arrhythmia had resolved irrespective of treatment (Figures 5A and 5B) ($p = 0.09$ and $p = 0.52$, respectively).

Antiarrhythmic treatment was safely discontinued between days 24 and 34 in all treated subjects that achieved electrical maturation without recrudescence of arrhythmia (Figure 5). Two treated and two untreated subjects (3 and 4 and 17 and 19, respectively) failed to mature electrically and exhibited significant arrhythmia at the end of study. In these four animals, heart rates were well controlled irrespective of treatment, and they survived until the study's completion. Average serum amiodarone was sub-therapeutic at 0.42 ± 0.12 μg/mL within 1 week of discontinuation (Figure S2).

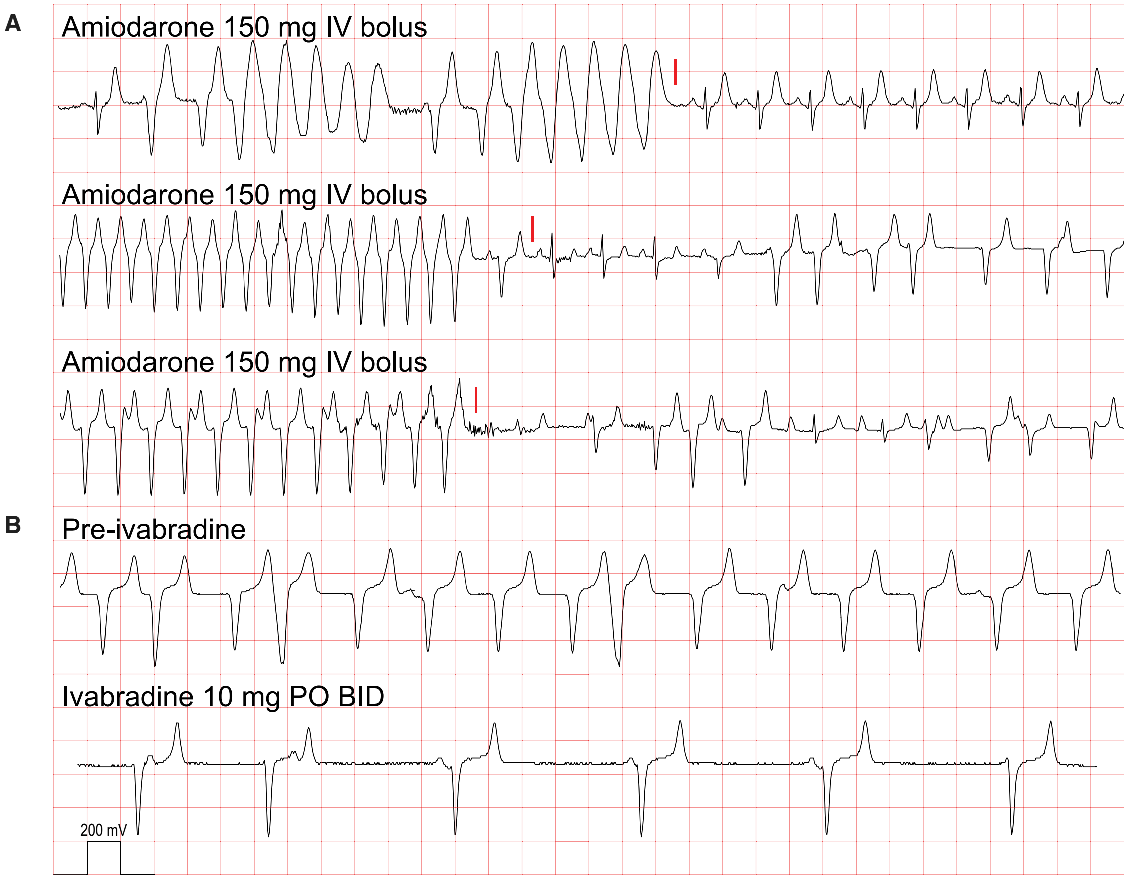

Figure 3. Acute effects of amiodarone and ivabradine on engraftment arrhythmia

Amiodarone was effective as an intravenous bolus to cardiovert engraftment arrythmia to normal sinus or a lower heart rate transiently in three separate instances (A) (red line). Ivabradine administered orally significantly slowed EA but did not cardiovert 2 days following initiation (B). These data supported a combined amiodarone and ivabradine antiarrhythmic strategy for rhythm and rate control of EA. One box vertical 200 mV, horizontal 0.2 s.

Percutaneous delivery of hESC-CMs in the infarcted porcine model

Catheter-based endocardial delivery of human ESC (hESC)-CMs was safe and effective in remuscularizing the infarcted porcine heart (Figure S3). No significant differences in myocardial infarct or cardiomyocyte graft sizes were observed between the treatment groups. The average infarct size for the treated and untreated cohorts were comparable at 10.3% ± 1% and 9.8% ± 1.8% of the left ventricle, respectively ($p = 0.82$). Graft size relative to infarct size was also comparable at 3.9% ± 1% and 3.2% ± 1.2% for treated and untreated subjects, respectively ($p = 0.64$). Delivery of hESC-CM successfully targeted the peri-infarct border zone and central ischemic regions as intended and resulted in discrete hPSC-CM grafts transplanted into host myocardium, as reported previously (Chong et al., 2014; Liu et al., 2018; Romagnuolo et al., 2019; Shiba et al., 2016). All grafts localized to the anterior, antero-septal, and antero-lateral walls and, as previously reported in pig (Romagnuolo et al., 2019), appeared structurally immature at early time points before 2 weeks post-transplantation with increasing maturity up to the end of study.

Graft interaction with the host Purkinje conduction system

The narrow complex tachycardia that resembles accelerated junctional rhythm (Figure 2) was not observed in our previous NHP studies (Chong et al., 2014; Liu et al., 2018) but was common in the minipig. Pigs are known to have an extensive Purkinje fiber network that extends transmurally throughout the ventricular myocardium, whereas in macaques and humans the Purkinje network is subendocardial (Garcia-Bustos et al., 2017; Panescu et al., 2014). We hypothesized that narrow complex VT resulted from graft automaticity conducting through intramural Purkinje fibers and propagating to the rest of the ventricle. Histology confirmed the mesh-like network of intramural Purkinje fibers throughout the minipig left

A Freedom from Primary Outcome

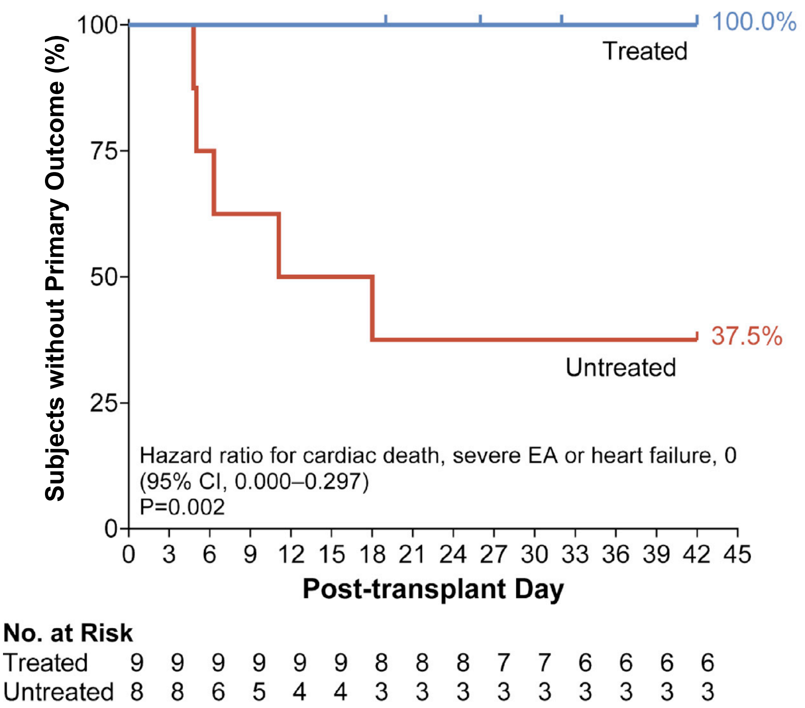

B Overall Survival

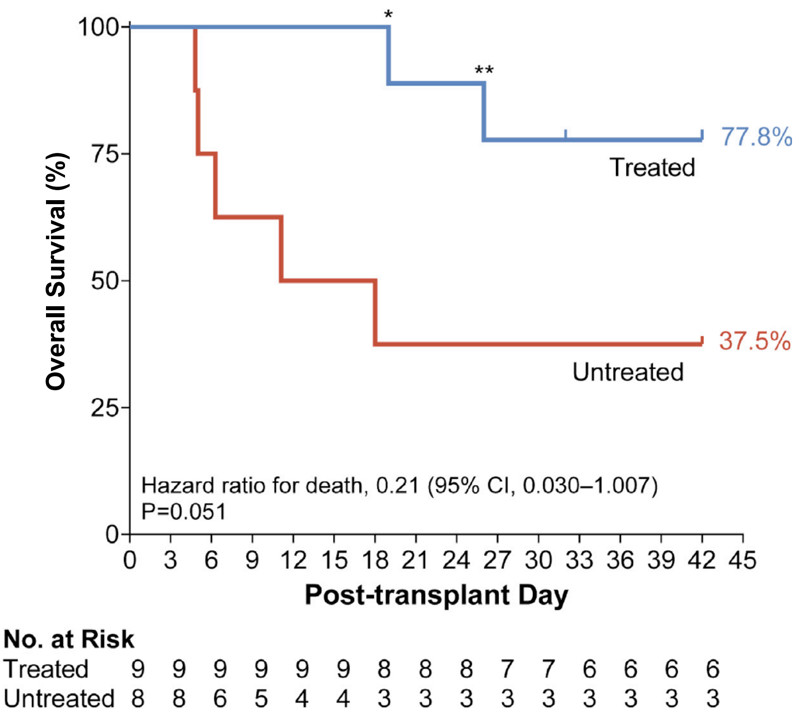

Figure 4. Antiarrhythmic treatment with amiodarone and ivabradine for engraftment arrhythmia in pig

(A) Kaplan-Meier curve for freedom from primary outcome of cardiac death, unstable EA or heart failure was significantly improved in the treated (n = 9) compared with the untreated cohort (n = 8, p = 0.002). Tic marks on the treatment line indicate non-cardiac death due to opportunistic infection (days 19 and 26) or a planned euthanasia (day 30).

(B) The Kaplan-Meier curve for overall survival shows statistically borderline improvement in the treated compared with the untreated cohort (p = 0.051). Each subject is an independent experiment. *Death due to *Pneumocystis* pneumonia. **Death due to porcine cytomegalovirus. CI, 95% confidence interval; No., number.

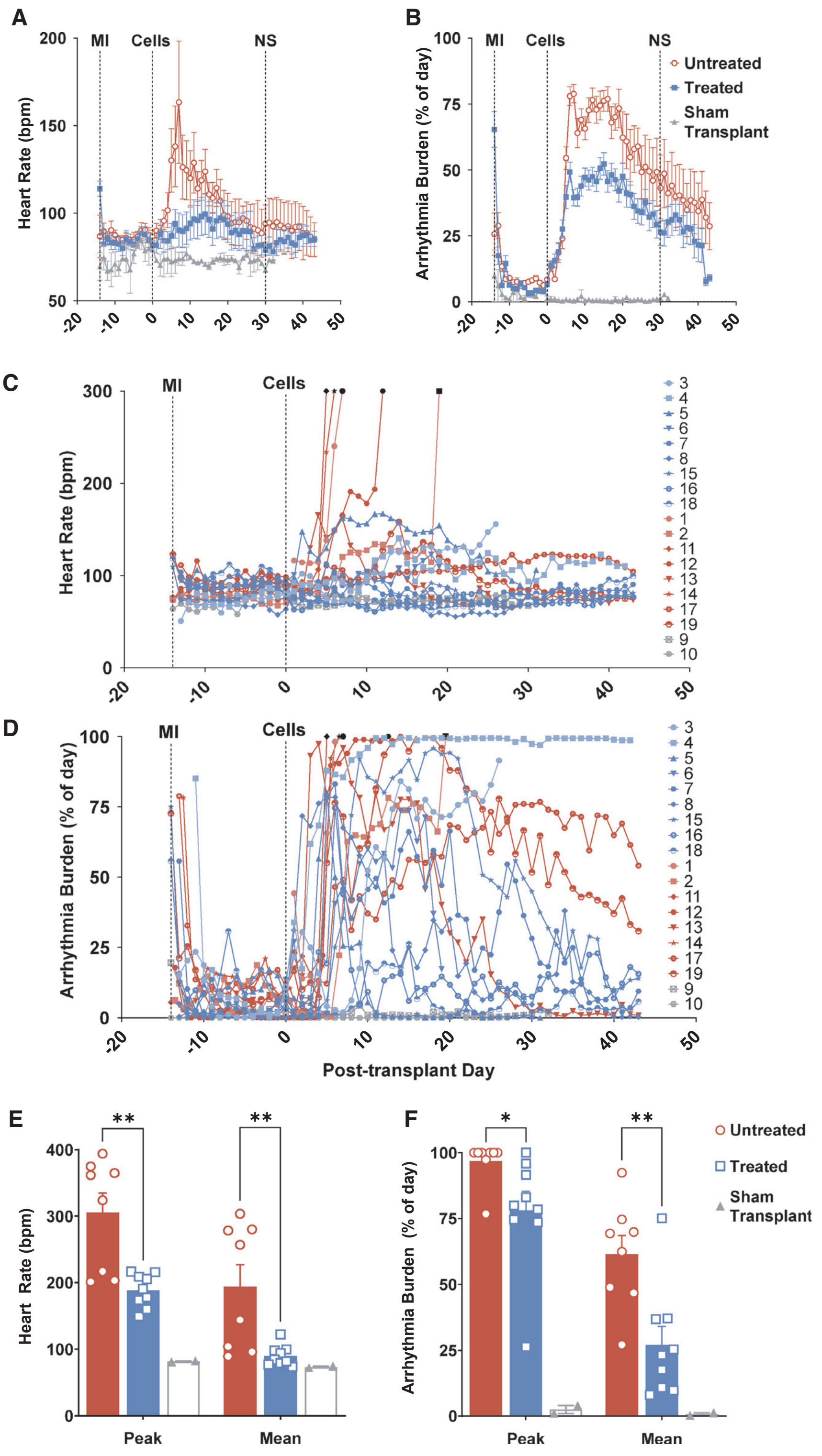

Figure 5. Effect of antiarrhythmic treatment on heart rate and arrhythmia burden
Pooled daily average heart rate (A) and pooled daily average arrhythmia burden (B) in treated (n = 9, blue) compared with untreated (n = 8, red) cohorts. The difference in heart rate or arrhythmia burden between treated and untreated cohorts was not significant (NS) by day 30 post-transplantation. Sham transplant (n = 2, gray) did not induce tachycardia or arrythmia. Data are represented as mean ± SEM and each subject is an independent experiment. Subject-level averaged daily heart rate (C) and arrhythmia burden (D) for antiarrhythmic treated (blue), untreated (red), and sham transplant (gray). Unexpected death or euthanasia are denoted by black symbols. Overall peak and mean daily heart rate (E) and overall peak and mean daily arrythmia burden (F) were significantly reduced in treated (blue) compared with untreated (red) cohorts. No tachycardia or arrhythmias were noted in the sham transplant control cohort. $^{*}p \le 0.05$, $^{**}p \le 0.01$.

ventricle (Figure S4A; Video S1). There were multiple examples of hESC-CM grafts in direct contact with these intramural branches of the Purkinje system (Figure 6; Video S2). We used connexin 40 (Cx40) immunostaining to specifically stain Purkinje fiber gap junctions (Garcia-Bustos et al., 2017; Pallante et al., 2010), and confirmed their identity by their reduced myofibril content and the absence of T tubules (Figure S4B). This supports the hypothesis that

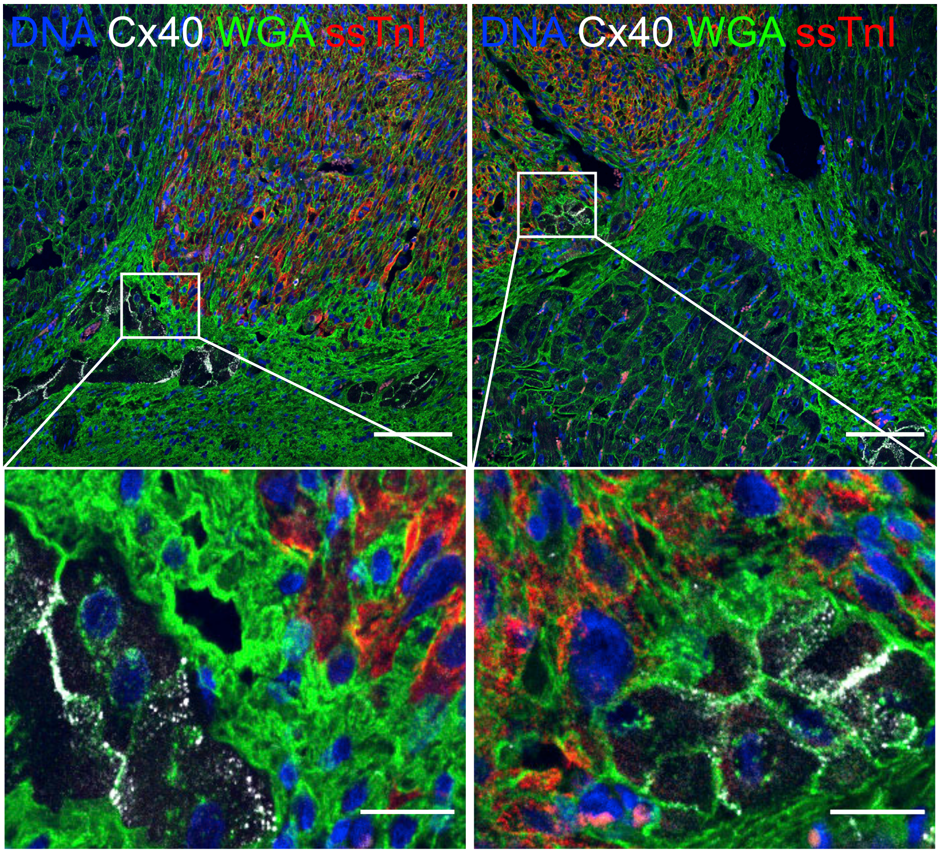

Figure 6. Transplanted hESC-CM grafts interact with a diffuse Purkinje conduction system in the porcine myocardium
An hPSC-cardiomyocyte graft 2 weeks post-transplantation, marked by human-specific slow skeletal cardiac troponin I (ssTnI) (red) (Bedada et al., 2014), interacts with Cx40-positive (white) Purkinje fibers. Wheat germ agglutinin (WGA) (green) delineates cardiac sarcolemma. Purkinje-transitional cell-graft (left panel) and direct Purkinje-graft (right panel) interactions are observed. Sale bars, 100 or 20 μm (magnified).

the pig's unique Purkinje network anatomy contributes to narrow complex EA.

DISCUSSION

Intramyocardial transplantation of hPSC-CMs is a promising strategy to remuscularize the infarcted heart and restore function (Nakamura and Murry, 2019). Such a therapy to prevent and treat heart failure would be a seminal advance in addressing a large unmet clinical need. Studies in large animals have demonstrated long-term efficacy but also defined a significant safety signal of generally transient but potentially fatal arrhythmias. As demonstrated in earlier studies (Liu et al., 2018; Romagnuolo et al., 2019; Shiba et al., 2016), EA is a predictable complication of cardiac remuscularization therapy for MI (Yu et al., 2019). In the NHPs, EA typically presents as a wide complex tachycardia with a variable electrical axis (Chong et al., 2014; Liu et al., 2018), and this was reproduced in the minipig recently by the Laflamme laboratory (Romagnuolo et al., 2019). Here, we further describe EA as polymorphic and interpret the changes in electrical axis as ectopy originating from different graft foci. Interestingly, in the pig we also observed a narrow complex VT that alternated with a wide complex tachycardia, a pattern not seen in the NHPs. Histology of native and grafted porcine myocardium supports the hypothesis that the wide complex beats originate from grafts interacting with the working cardiac myocytes with slow conduction, and that the narrow complex beats originate when grafts interact with the intramural Purkinje fibers that diffusely permeate the porcine heart (Garcia-Bustos et al., 2017; Panescu et al., 2014).

All 17 subjects transplanted with 500×10^6 hESC-CMs demonstrated significant burden of arrhythmia that, while typically transient, was associated with high mortality in pigs. We observed higher morbidity and mortality related to EA than the recent study by Laflamme and colleagues

(Romagnuolo et al., 2019), perhaps reflecting differences in our animal model including the use of Yucatan minipigs, percutaneous cell delivery, or our cell product. Our experience with this model suggests two primary mechanisms of cardiac morbidity. Firstly, rapid EA >350 bpm often degenerates to fatal VF and, secondly, heart failure commonly ensues in pigs with chronic tachycardia >230 bpm (Chow et al., 1990). Consequently, our primary endpoint included these parameters to limit excessive mortality in our antiarrhythmic trial.

Combined antiarrhythmic treatment with baseline amiodarone and adjunctive ivabradine safely prevented the combined primary endpoint of cardiac death, unstable EA, and heart failure in all treated subjects, indicating that the risk of EA may be mitigated through pharmacology. Treatment was associated with significantly decreased peak tachycardia and arrhythmia. Once subjects experienced sustained improvement in arrhythmia burden, termed electrical maturation, antiarrhythmic therapy was successfully withdrawn in all subjects. Thus, short-term amiodarone and ivabradine treatment promoted electrical stability until the grafts became less arrhythmogenic.

The mechanism of benefit for our antiarrhythmic treatment may be related to suppression of automaticity, reducing both heart rate and arrhythmia burden. The drugs were particularly beneficial during the early phase of EA, which carries the greatest risk of deterioration to VF. Electrophysiological studies performed by our group and the Laflamme laboratory in NHPs (Liu et al., 2018) and pig (Romagnuolo et al., 2019), respectively, suggests that the etiology of EA is increased focal automaticity, rather than macro-reentry typically observed with clinical VT (Josephson et al., 1984). In our study, cardioversion was unsuccessful in terminating EA. As EA became unstable in the untreated animals, heart rates rapidly accelerated to >350 bpm, and we cannot exclude the possibility that this escalation could have a distinct mechanism, e.g., automaticity leading to reentry. This may explain why treatment successfully suppressed unstable and fatal arrhythmias but was unable to prevent EA altogether.

The efficacy of ivabradine to rate control EA suggests that its pharmacologic target, the I_f current carried by the HCN4 channel, which is highly expressed in immature cardiomyocytes and hPSC-CMs (Karbassi et al., 2020), may be an important mediator. Ivabradine, by itself, never abrogated EA, suggesting that, while the I_f current can accelerate the rate of EAs, I_f is not the current responsible for generating the ventricular automaticity triggered by engraftment. In contrast, amiodarone reduced the burden of EA chronically and clearly restored sinus rhythm in some acute infusion experiments (Figure 3). Although classified principally as a K^+ channel blocker (class III), amiodarone is well known also to antagonize Na^+ channels, Ca^{2+} channels, and β-adrenergic receptors (Waks and Zimetbaum, 2017). Thus, it is difficult to gain insights into the mechanism of EA from amiodarone's efficacy. The disappearance of EA coincides with maturation of the stem cell-derived graft (Chong et al., 2014; Kadota et al., 2017; Liu et al., 2018), and we with others have hypothesized that the window of arrhythmogenicity may reflect a period of *in vivo* graft maturation before reaching a state more similar to host myocardium (Guo and Pu, 2020; Ichimura et al., 2020; Kannan and Kwon, 2020; Karbassi et al., 2020; Marchiano et al., 2019; Maroli and Braun, 2020). Additional strategies such as promoting maturation before transplantation, gene editing, and modulating host/cell interaction may provide additional means of arrhythmia control and a comprehensive protocol invoking multiple complementary mechanisms of action may ultimately be necessary to ensure safety. Further investigation of the etiology of EA would be accelerated by the development of higher throughput *in vivo*, *ex vivo*, *in vitro*, and/or *in silico* platforms to perform genetic, pharmacological, electrophysiological, and/or modeling studies before phenotyping in large animal models.

EA is the most significant barrier to clinical translation of cardiac remuscularization therapy. The natural history of EA emerging from the NHPs and more recent porcine data suggests that, once EA resolves, there is low risk for further arrhythmia. This study provides a proof-of-concept that clinically relevant antiarrhythmic drug treatment can successfully suppress fatal arrhythmias and control tachycardia to achieve electrical quiescence. This could be an important tool toward reaching an acceptable safety profile for clinical development.

While this study demonstrates that EA is responsive to pharmacologic suppression, there are several limitations. Notably, our proposed regimen did not completely abrogate EA and several treated subjects experienced significant, but not fatal, arrhythmia. As such, adjunctive methods may be desirable to further mitigate risk from EA to patients. Possible strategies may include promoting maturation and modification of hESC-CMs as well as dosing and delivery protocols to be less arrhythmogenic. This study would also benefit from longer follow-up to establish the long-term effectiveness of EA mitigation. We did not randomize enrollment of animals or assess whether sex is a biological variable. Although we took pains to administer clinically relevant doses of amiodarone and ivabradine, we cannot exclude the possibility that EA in itself is dependent on the dose of cells transplanted. The dose utilized is comparable with that utilized to demonstrate long-term function benefit in NHPs (500×10^6 versus 750×10^6 hESC-CMs, respectively) (Liu et al., 2018), but dosing studies have not been reported. Future studies will also ideally include functional endpoints to determine

mechanical efficacy with background guideline-directed medical therapy, such as inhibitors of the renin-angiotensin-aldosterone and β-adrenergic systems.

In this study utilizing a porcine infarction model of cardiac remuscularization therapy, EA was universally observed and associated with significant mortality. Chronic amiodarone treatment combined with adjunctive ivabradine successfully prevented the combined primary endpoint of cardiac death, unstable EA, and heart failure. Overall survival was significantly improved with antiarrhythmic treatment and associated with heart rate and rhythm control. The mechanisms of EA remain incompletely understood and merit concerted scientific inquiry.

EXPERIMENTAL PROCEDURES

hESC-CM production

These studies were approved by the University of Washington Stem Cell Research Oversight Committee. Two lines of hESCs were used in this study. Initial subjects received H7 (WiCell)-derived cardiomyocytes that were cultured, expanded, and differentiated in suspension culture format by collaborators at the Center for Applied Technology Development at the City of Hope in California, all as described previously (Chen et al., 2015; Chong et al., 2014; Liu et al., 2018). Most subjects received RUES2 (Rockefeller University)-derived cardiomyocytes produced in our laboratory in stirred suspension culture format. In brief, RUES2 hESCs were cultured to form aggregates and were expanded in commercially available medium (Essential 8, Gibco). For cardiac differentiation, suspension-adapted pluripotent aggregates were induced to differentiate in RPMI-1640, MCDB-131, or M199 supplemented with B-27 (all from Gibco) or serum albumin, by timed use of small-molecule GSK 3 inhibitor, CHIR 99021 (days 0 to 1), and Wnt/β-catenin signal pathway inhibitors (days 2 to 4) (Tocris). Twenty-four hours before cryopreservation, RUES2 hESC-CMs were heat shocked to enhance their survival after harvest, cryopreservation, thaw, and transplantation. Cardiomyocyte aggregates were dissociated by treatment with Liberase TH (Fisher) and TrypLE (Gibco) and were cryopreserved in CryoStor CS10 (STEMCELL Technologies) supplemented with 10 μM Y-27632 (STEMCELL Technologies) using a controlled-rate liquid nitrogen freezer. Approximately 3 h before transplantation, cryopreserved hESC-CMs were removed from cryogenic storage (-150°C to -196°C) and thawed in a 37°C water bath (2 min ± 30 s). RPMI-1640 supplemented with B-27 and ≥200 Kunitz units/mL DNase I (Millipore) was added to the cell suspension to dilute the cryopreservation media. Subsequent wash steps were done using RPMI-1640 basal medium in progressively smaller volumes to concentrate the cell suspension. For the last centrifugation step, the cell pellet was resuspended in a sufficient volume of RPMI-1640 to achieve a target cell density for injection of $\sim 3 \times 10^9$ cells/mL in 1.6 mL. The final volume of the cell suspension was determined by the results of a count sampled before the final centrifugation step. Cell counts were performed as described previously to achieve a final total dose of 500×10^6 live cells per transplant.

Study design

The objective of this study was to identify a pharmacological regimen to attenuate arrhythmias following cardiac remuscularization therapy. This study was designed in two phases: the first to observe the natural history of EA in the minipig model and screen various antiarrhythmic agents for possible efficacy, and the second to rigorously test for efficacy of selected candidates (Figure 1). All protocols were approved and conducted in accordance with the University of Washington (UW) Office of Animal Welfare and the Institutional Animal Care and Use Committee. All subjects were 30–40 kg castrated male Yucatan minipigs between 6 and 13 months of age (Premier BioSource). In phase 1, nine subjects underwent cardiac remuscularization therapy with 500×10^6 hESC-CMs delivered by direct surgical trans-epicardial injections, or later by percutaneous trans-endocardial injections (Table 1). The first four subjects (one non-infarcted and three infarcted) were followed to learn the natural history of EA and establish clinical endpoints and parameters for the phase 2 trial. The subsequent five subjects underwent systematic dosing with antiarrhythmic agents with continuous electrocardiography (ECG) monitoring to determine effect on rhythm and rate (Table S1, supplemental information). In brief, subjects were administered serial trials of antiarrhythmics and observed for acute response by continuous ECG monitoring. All agents were tested in at least two subjects. Details are provided in the supplemental information.

Among the nine subjects in phase 1, we observed high mortality, with six out of nine experiencing VF or tachycardia-induced heart failure requiring euthanasia. VF typically occurred within the first week of transplantation and following frequent episodes of unstable EA >350 bpm, and tachycardia-induced heart failure requiring euthanasia was characterized by chronically elevated heart rates >150 bpm for 1–2 weeks.

In phase 2, we conducted a two-drug antiarrhythmic study with amiodarone and ivabradine, enrolling an additional 17 subjects (9 treated, 8 untreated) that underwent MI and percutaneous transplantation with hESC-CMs at 2 weeks post-MI. Two additional subjects underwent MI with sham vehicle injection to serve as sham transplant controls (Figures 1 and S1). The primary endpoint was prespecified as combined cardiac death (either spontaneous death from arrhythmia or heart failure, or clinically directed euthanasia necessitated by sustained tachycardia >350 bpm or signs of heart failure). Prespecified secondary endpoints were suppression of tachycardia, percent time in arrhythmia (arrhythmia burden) and resolution of arrhythmia, termed "electrical maturation" and defined as arrhythmia burden <25% for 48 consecutive hours. Antiarrhythmic therapy was discontinued after electrical maturation or at post-transplantation day 30, whichever was earlier. To prevent tachycardia-induced cardiomyopathy, we titrated ivabradine treatment to maintain target heart rate <150 bpm. Based on early experience that tachycardia >350 bpm often degenerated to VF, subjects were euthanized humanely if heart rates >350 bpm were reached. Continuous telemetric ECG was monitored for 8 weeks in total (2 weeks post-MI and 6 weeks post-transplantation). Of note, subjects 1 and 2 (untreated) and 3 and 4 (treated) received H7 hESC-CMs and subjects 1–3 were transplanted surgically before adopting percutaneous delivery. Subject 5 exhibited early electrical maturation on day 12

and was euthanized on day 31 before extending the study duration to 6 weeks post-transplantation for extended treatment washout and monitoring for all subjects.

Cardiac remuscularization therapy

MI was induced percutaneously 2 weeks before cell transplantation (supplemental information). Cell transplantation for our three initial subjects (1–3) was performed by direct trans-epicardial injection into the peri-infarction region as previously described for NHPs with minor modification (Liu et al., 2018) and described in the supplemental information. All subsequent subjects (4–19) received cell transplantation via percutaneous trans-endocardial injection using the NOGA-MyoStar platform (BioSense Webster). In brief, trans-epicardial injection via partial median sternotomy was performed to expose the infarcted anterior left ventricle. Purse-string sutures were preplaced at five discrete locations subtended by the LAD, targeting the central ischemic region (2/5) and lateral border zones identified by gross inspection and adjudicated by consensus of the surgical team. After cinching the purse-string tightly around the needle, three injections of 100 μL each were performed by partial withdrawal and lateral repositioning, for a total of 15 injections to deliver a total dose of 500×10^6 hESC-CMs. Percutaneous trans-endocardial injection using the NOGA-MyoStar platform (BioSense Webster) was performed by first mapping the infarct region in the left ventricle, and then deliver 16 discrete endocardial injections of 100 μL each for a total dose of 500×10^6 hESC-CMs. Injections were only performed with excellent location and loop stability, ST-segment elevation, and the presence of premature ventricular contraction with needle insertion in an appropriate location by electroanatomical map and unipolar voltage. Two-thirds of injections were placed into the peri-infarct border zone defined by a unipolar voltage of 5–7.5 mV, and the remaining one-third into the central ischemic region, defined as unipolar voltage of <5 mV. Two subjects (9 and 10) were infarcted as per protocol but received sham injections of RPMI-1640 vehicle without cells to serve as sham transplant controls.

Immunosuppression therapy

All three cohorts received a three-drug immunosuppression regimen to prevent xenograft rejection, as described previously with modification (Liu et al., 2018). For our initial regimen (subjects 1–6), 5 days before cell transplantation, oral cyclosporine A was started to maintain a serum trough level of >400 ng/mL (approximately 250–1,000 mg twice daily) for the duration of the study. Two days before transplantation, oral methylprednisolone was started at 3 mg/kg for 2 weeks and then titrated down to 1.5 mg/kg for the remainder of the study. On the day of transplantation, Abatacept (CTLA4-Ig, Bristol-Myers Squibb), 12.5 mg/kg, was administered intravenously and dosed every 2 weeks thereafter. Due to complications related to immunosuppression (principally porcine cytomegalovirus and *Pneumocystis* pneumonia), the cyclosporine A trough level was decreased to >300 ng/mL and the methylprednisolone reduced to 1.0 mg/kg for subjects 7–19 without histologic evidence of rejection. Prophylactic oral cephalexin was administered for all subjects to prevent infection of the indwelling central venous catheter. Prophylactic sulfamethoxazole/trimethoprim was added after subject 3 developed *Pneumocystis* pneumonia. Prophylactic valganciclovir and probiotics were added after activation of endogenous porcine cytomegalovirus (CMV) was found in subject 6. Further complications related to CMV were not observed in subsequent subjects with prophylaxis.

Antiarrhythmic treatment

The treated cohort was loaded with oral amiodarone 1,000–1,200 mg orally twice daily starting 7 days before cell transplantation followed by a maintenance dose of 400–1,000 mg orally twice daily to maintain a steady-state plasma level of 1.5–4.0 μg/mL (Figure S2). Ivabradine was started at 2.5 mg orally twice daily when sustained tachycardia reached ≥150 bpm and titrated every 3 days up to 15 mg twice daily for a goal heart rate of <125 bpm. All but one subject (1) required adjunctive ivabradine for additional heart rate control. Antiarrhythmics were discontinued after electrical maturation was achieved or at post-transplantation day 30, whichever was earlier, to allow for treatment washout and assess for recrudescence of arrhythmia. All subjects tolerated the antiarrhythmic regimen without complication. Untreated and sham transplant control subjects did not receive antiarrhythmic agents following the MI procedure, but otherwise received all immunosuppression and standard care.

Amiodarone drug monitoring

Liquid chromatography-mass spectrometry was used to monitor steady-state serum levels of amiodarone in the minipig model and guide oral dosing to ensure efficacy and avoid dose-related toxicity. A target serum level of 1.5–4.0 μg/mL was extrapolated from previous human pharmacokinetic studies (Mostow et al., 1984; Staubli et al., 1983). Elimination kinetics after discontinuation of oral amiodarone therapy were also studied by obtaining weekly trough concentrations in six subjects (6, 7, 8, 13, 14, and 16) (Figure S2).

ECG analysis

Telemetric ECG was continuously monitored in real time from the time of MI to detect the primary endpoint of cardiac death or unstable EA. Automated quantification of heart rate and arrhythmia burden was performed offline by a board-certified cardiologist using the ecgAUTO 3.3.5.10 software package (EMKA Technologies). Arrhythmia was defined as an ectopic beat (e.g., premature ventricular contraction) or rhythm (e.g., idioventricular rhythm, VT). EA was typically observed as sustained and non-sustained ventricular tachyarrhythmia of varying rates and morphologies, but also included slow and narrow complex ectopic rhythms (Figure 2). Heart rate and arrhythmia burden were quantified continuously for phase 1 subjects. For phase 2 subjects, two continuous minutes every 5 min (40% of total rhythm was counted) was analyzed and presented as daily averages.

Statistical analysis

Statistical analyses and graphing were performed using Prism 8.4.2 software (GraphPad) and Stata 15 (StataCorp, College Station, TX). Data are presented as mean ± standard error of the mean (SEM). Comparisons were performed using the Mann-Whitney test with significance threshold of $p < 0.05$. The sample size to demonstrate a difference in mortality rate of 67% (untreated group) versus 0%

(treatment group), with alpha = 0.05 and 90% power, was estimated to be 8 per cohort. Kaplan-Meier plots show survival curves for the primary endpoint of cardiac death, unstable EA or heart failure, and for all-cause mortality. Cox proportional regression models were used to estimate the hazard ratio between the two treatment groups for the primary outcome and for mortality. Significance is based on the likelihood ratio test and confidence intervals on hazard ratio were computed by inverting the likelihood test, based on varying the offset term in the stcox procedure in Stata.

Histologic analysis

Histological studies were carried out as detailed previously with modification (Chong et al., 2014; Liu et al., 2018). In brief, paraformaldehyde-fixed hearts were dissected to remove the atria and right ventricle before short-axis cross-sections were cut at 2.5 mm intervals. The weights of the whole heart, left ventricle, and each slice were obtained before further partition into tissue cassettes. The tissue then was processed, embedded in paraffin, and 4 μm sections were cut for staining. For morphometry, infarct regions were identified by Picrosirius red staining; human graft was identified by anti-human cardiac troponin T (Invitrogen, MA5-12960), stained using avidin-biotin reaction (ABC Kit, VectorLabs), followed by chromogenic detection via diaminobenzidine (Sigmafast, Sigma Life Science) (Figure S3). The slides were digitized using a whole slide scanner (Nanozoomer, Hamamatsu), and the images were viewed and exported with NDP.view 2.6.13 (Hamamatsu). Areas of infarct and graft were analyzed using a custom-written algorithm in the ImageJ open source software platform (Schindelin et al., 2015). In brief, after extracting images in TIFF format (Deroulers et al., 2013), the image foreground was segmented by a threshold derived from the distribution in brightness of its pixels, resulting in a binary mask that delineates the imaged tissue section. Subsequent color de-convolution by thresholding hue, brightness, and saturation allowed segmentation of regions stained by Picrosirius red stain or areas immunolabeled for human cardiac troponin T. To separate scar from diffuse fibrosis, a cutoff for particle size was applied. Infarct size and graft size were calculated (percent area × block weight), summed for the entire ventricle, and expressed as a percentage of left ventricular mass or infarct mass, respectively. Please see the supplemental information for Purkinje fiber staining.

SUPPLEMENTAL INFORMATION

Supplemental information can be found online at https://doi.org/10.1016/j.stemcr.2021.08.005.

AUTHOR CONTRIBUTIONS

K.N. conceived the study, led experimental design, performed surgical and percutaneous procedures, analyzed and interpreted data, and wrote the manuscript. L.E.N. assisted with the conception, experimental design, and execution of the study, performed surgical and percutaneous procedures, supervised veterinary care, contributed to data analysis, and edited the manuscript. X.Y. performed histologic analysis and edited the manuscript. G.J.W. developed the experimental procedures, performed histologic analysis, and contributed to the manuscript. D.E.-N. performed histologic analysis and contributed to the manuscript. H.T. assisted with surgical and percutaneous procedures, assisted with experimental design, and performed histologic analysis. S.D. assisted with surgical and percutaneous procedures and performed histologic analysis. F.A.K. cultured and characterized the hESC-CM cell product. A.J. cultured and characterized the hESC-CM cell product. A.F.-T. cultured and characterized the hESC-CM cell product. D.S.N. assisted with experimental design and writing the manuscript. S.M. assisted with experimental design and edited the manuscript. A.B. assisted with experimental design and edited the manuscript. M.R.R. assisted with the study's conception, experimental design, and execution. K.C. performed statistical analysis and contributed to the manuscript. R.T. supervised histologic analysis and development of experimental procedure. H.R. assisted with the study's conception, experimental design, and execution. L.P. assisted with the study's conception, experimental design, and execution. B.C.K. assisted with the study's conception and experimental design. S.K. with assisted the study's experimental design and execution and supervised cell manufacturing. R.S.T. assisted with the study's conception, experimental design, and execution, supervised cell manufacturing, and obtained research funding. W.R.M. assisted with the study's conception, experimental design, and execution, and obtained research funding. C.E.M. conceived and supervised the study, obtained research funding, and contributed to data analysis and writing of the manuscript.

CONFLICTS OF INTEREST

K.N., M.R.R., B.C.K., and W.R.M. are advisors to Sana Biotechnology. D.E.-N., H.T., S.D., F.A.K., A.J., A.F.-T., D.S.N., S.K., R.S.T., and C.E.M. are employees of Sana Biotechnology. C.E.M. is a scientific founder and equity holder of Sana Biotechnology. The remaining authors have nothing to disclose. These studies were supported by the UW Medicine Heart Regeneration Program, the Washington Research Foundation, and a gift from Mike and Lynn Garvey (all Seattle, WA). This work also was supported in part by NIH grants R01HL128362, and a grant from the Fondation Leducq Transatlantic Network of Excellence (Boston, MA; to C.E.M.) and the Bruce-Laughlin Research Fellowship (Seattle, WA; to K.N.).

ACKNOWLEDGMENTS

We are indebted to Emily Spaulding, Gary Fye, Piper M. Treuting, Thea Brabb, and colleagues in the UW Department of Comparative Medicine for their exceptional veterinary care and consultation. We acknowledge the Center for Applied Technology Development at the City of Hope (Duarte, CA) for providing H7 hESC-CM used for our studies and use of these cells was funded in part through the National Heart, Lung, and Blood Institute Production Assistance for Cell Therapies (PACT) (Bethesda, MD). We thank the Garvey Imaging Core at UW for assistance with microscopy.

Received: April 21, 2021
Revised: August 9, 2021
Accepted: August 10, 2021
Published: September 9, 2021

REFERENCES

Bedada, F.B., Chan, S.S., Metzger, S.K., Zhang, L., Zhang, J., Garry, D.J., Kamp, T.J., Kyba, M., and Metzger, J.M. (2014). Acquisition of a quantitative, stoichiometrically conserved ratiometric marker of maturation status in stem cell-derived cardiac myocytes. Stem Cell Reports *3*, 594–605. https://doi.org/10.1016/j.stemcr.2014.07.012.

Caspi, O., Huber, I., Kehat, I., Habib, M., Arbel, G., Gepstein, A., Yankelson, L., Aronson, D., Beyar, R., and Gepstein, L. (2007). Transplantation of human embryonic stem cell-derived cardiomyocytes improves myocardial performance in infarcted rat hearts. J. Am. Coll. Cardiol. *50*, 1884–1893. https://doi.org/10.1016/j.jacc.2007.07.054.

Chen, V.C., Ye, J., Shukla, P., Hua, G., Chen, D., Lin, Z., Liu, J.C., Chai, J., Gold, J., Wu, J., et al. (2015). Development of a scalable suspension culture for cardiac differentiation from human pluripotent stem cells. Stem Cell Res *15*, 365–375. https://doi.org/10.1016/j.scr.2015.08.002.

Chong, J.J., Yang, X., Don, C.W., Minami, E., Liu, Y.W., Weyers, J.J., Mahoney, W.M., Van Biber, B., Cook, S.M., Palpant, N.J., et al. (2014). Human embryonic-stem-cell-derived cardiomyocytes regenerate non-human primate hearts. Nature *510*, 273–277. https://doi.org/10.1038/nature13233.

Chow, E., Woodard, J.C., and Farrar, D.J. (1990). Rapid ventricular pacing in pigs: an experimental model of congestive heart failure. Am. J. Physiol. *258*, H1603–H1605. https://doi.org/10.1152/ajpheart.1990.258.5.H1603.

Deroulers, C., Ameisen, D., Badoual, M., Gerin, C., Granier, A., and Lartaud, M. (2013). Analyzing huge pathology images with open source software. Diagn. Pathol. *8*, 92. https://doi.org/10.1186/1746-1596-8-92.

Eschenhagen, T., Bolli, R., Braun, T., Field, L.J., Fleischmann, B.K., Frisen, J., Giacca, M., Hare, J.M., Houser, S., Lee, R.T., et al. (2017). Cardiomyocyte regeneration: a consensus statement. Circulation *136*, 680–686. https://doi.org/10.1161/CIRCULATIONAHA.117.029343.

Garcia-Bustos, V., Sebastian, R., Izquierdo, M., Molina, P., Chorro, F.J., and Ruiz-Sauri, A. (2017). A quantitative structural and morphometric analysis of the Purkinje network and the Purkinje-myocardial junctions in pig hearts. J. Anat. *230*, 664–678. https://doi.org/10.1111/joa.12594.

Guo, Y., and Pu, W.T. (2020). Cardiomyocyte maturation: new phase in development. Circ. Res. *126*, 1086–1106. https://doi.org/10.1161/CIRCRESAHA.119.315862.

Ichimura, H., Kadota, S., Kashihara, T., Yamada, M., Ito, K., Kobayashi, H., Tanaka, Y., Shiba, N., Chuma, S., Tohyama, S., et al. (2020). Increased predominance of the matured ventricular subtype in embryonic stem cell-derived cardiomyocytes in vivo. Sci. Rep. *10*, 11883. https://doi.org/10.1038/s41598-020-68373-9.

Josephson, M., Marchlinski, F., Buxton, A., Waxman, H., Doherty, J., Kienzle, M., and Falcone, R. (1984). Electrophysiologic basis for sustained ventricular tachycardia: role of reentry. In Tachycardias: Mechanisms, Diagnosis, Treatment (Lea and Febiger), pp. 305–323.

Kadota, S., Pabon, L., Reinecke, H., and Murry, C.E. (2017). In vivo maturation of human induced pluripotent stem cell-derived cardiomyocytes in neonatal and adult rat hearts. Stem Cell Reports *8*, 278–289. https://doi.org/10.1016/j.stemcr.2016.10.009.

Kannan, S., and Kwon, C. (2020). Regulation of cardiomyocyte maturation during critical perinatal window. J. Physiol. *598*, 2941–2956. https://doi.org/10.1113/JP276754.

Karbassi, E., Fenix, A., Marchiano, S., Muraoka, N., Nakamura, K., Yang, X., and Murry, C.E. (2020). Cardiomyocyte maturation: advances in knowledge and implications for regenerative medicine. Nat. Rev. Cardiol. *17*, 341–359. https://doi.org/10.1038/s41569-019-0331-x.

Laflamme, M.A., Chen, K.Y., Naumova, A.V., Muskheli, V., Fugate, J.A., Dupras, S.K., Reinecke, H., Xu, C., Hassanipour, M., Police, S., et al. (2007). Cardiomyocytes derived from human embryonic stem cells in pro-survival factors enhance function of infarcted rat hearts. Nat. Biotechnol. *25*, 1015–1024. https://doi.org/10.1038/nbt1327.

Lelovas, P.P., Kostomitsopoulos, N.G., and Xanthos, T.T. (2014). A comparative anatomic and physiologic overview of the porcine heart. J. Am. Assoc. Lab Anim. Sci. *53*, 432–438.

Liu, Y.W., Chen, B., Yang, X., Fugate, J.A., Kalucki, F.A., Futakuchi-Tsuchida, A., Couture, L., Vogel, K.W., Astley, C.A., Baldessari, A., et al. (2018). Human embryonic stem cell-derived cardiomyocytes restore function in infarcted hearts of non-human primates. Nat. Biotechnol. *36*, 597–605. https://doi.org/10.1038/nbt.4162.

Marchiano, S., Bertero, A., and Murry, C.E. (2019). Learn from your elders: developmental biology lessons to guide maturation of stem cell-derived cardiomyocytes. Pediatr. Cardiol. *40*, 1367–1387. https://doi.org/10.1007/s00246-019-02165-5.

Maroli, G., and Braun, T. (2020). The long and winding road of cardiomyocyte maturation. Cardiovasc. Res. *117*, 712–726. https://doi.org/10.1093/cvr/cvaa159.

Mostow, N.D., Rakita, L., Vrobel, T.R., Noon, D.L., and Blumer, J. (1984). Amiodarone: correlation of serum concentration with suppression of complex ventricular ectopic activity. Am. J. Cardiol. *54*, 569–574. https://doi.org/10.1016/0002-9149(84)90250-9.

Nakamura, K., and Murry, C.E. (2019). Function follows form—a review of cardiac cell therapy. Circ. J. *83*, 2399–2412. https://doi.org/10.1253/circj.CJ-19-0567.

Pallante, B.A., Giovannone, S., Fang-Yu, L., Zhang, J., Liu, N., Kang, G., Dun, W., Boyden, P.A., and Fishman, G.I. (2010). Contactin-2 expression in the cardiac Purkinje fiber network. Circ. Arrhythm Electrophysiol. *3*, 186–194. https://doi.org/10.1161/CIRCEP.109.928820.

Panescu, D., Kroll, M., and Brave, M. (2014). Limitations of animal electrical cardiac safety models. Annu. Int. Conf. IEEE Eng. Med. Biol. Soc. *2014*, 6483–6486. https://doi.org/10.1109/EMBC.2014.6945113.

Romagnuolo, R., Masoudpour, H., Porta-Sanchez, A., Qiang, B., Barry, J., Laskary, A., Qi, X., Masse, S., Magtibay, K., Kawajiri, H., et al. (2019). Human embryonic stem cell-derived cardiomyocytes regenerate the infarcted pig heart but induce ventricular tachyarrhythmias. Stem Cell Reports *12*, 967–981. https://doi.org/10.1016/j.stemcr.2019.04.005.

Schindelin, J., Rueden, C.T., Hiner, M.C., and Eliceiri, K.W. (2015). The ImageJ ecosystem: an open platform for biomedical image analysis. Mol. Reprod. Dev. *82*, 518–529. https://doi.org/10.1002/mrd.22489.

Shiba, Y., Fernandes, S., Zhu, W.Z., Filice, D., Muskheli, V., Kim, J., Palpant, N.J., Gantz, J., Moyes, K.W., Reinecke, H., et al. (2012). Human ES-cell-derived cardiomyocytes electrically couple and suppress arrhythmias in injured hearts. Nature *489*, 322–325. https://doi.org/10.1038/nature11317.

Shiba, Y., Filice, D., Fernandes, S., Minami, E., Dupras, S.K., Biber, B.V., Trinh, P., Hirota, Y., Gold, J.D., Viswanathan, M., and Laflamme, M.A. (2014). Electrical integration of human embryonic stem cell-derived cardiomyocytes in a guinea pig chronic infarct model. J. Cardiovasc. Pharmacol. Ther. *19*, 368–381. https://doi.org/10.1177/1074248413520344.

Shiba, Y., Gomibuchi, T., Seto, T., Wada, Y., Ichimura, H., Tanaka, Y., Ogasawara, T., Okada, K., Shiba, N., Sakamoto, K., et al. (2016). Allogeneic transplantation of iPS cell-derived cardiomyocytes regenerates primate hearts. Nature *538*, 388–391. https://doi.org/10.1038/nature19815.

Staubli, M., Bircher, J., Galeazzi, R.L., Remund, H., and Studer, H. (1983). Serum concentrations of amiodarone during long term therapy. Relation to dose, efficacy and toxicity. Eur. J. Clin. Pharmacol. *24*, 485–494. https://doi.org/10.1007/bf00609891.

van der Spoel, T.I., Jansen of Lorkeers, S.J., Agostoni, P., van Belle, E., Gyongyosi, M., Sluijter, J.P., Cramer, M.J., Doevendans, P.A., and Chamuleau, S.A. (2011). Human relevance of pre-clinical studies in stem cell therapy: systematic review and meta-analysis of large animal models of ischaemic heart disease. Cardiovasc. Res. *91*, 649–658. https://doi.org/10.1093/cvr/cvr113.

van Laake, L.W., Passier, R., Monshouwer-Kloots, J., Verkleij, A.J., Lips, D.J., Freund, C., den Ouden, K., Ward-van Oostwaard, D., Korving, J., Tertoolen, L.G., et al. (2007). Human embryonic stem cell-derived cardiomyocytes survive and mature in the mouse heart and transiently improve function after myocardial infarction. Stem Cell Res. *1*, 9–24. https://doi.org/10.1016/j.scr.2007.06.001.

Velagaleti, R.S., Pencina, M.J., Murabito, J.M., Wang, T.J., Parikh, N.I., D'Agostino, R.B., Levy, D., Kannel, W.B., and Vasan, R.S. (2008). Long-term trends in the incidence of heart failure after myocardial infarction. Circulation *118*, 2057–2062. https://doi.org/10.1161/CIRCULATIONAHA.108.784215.

Waks, J.W., and Zimetbaum, P. (2017). Antiarrhythmic drug therapy for rhythm control in atrial fibrillation. J. Cardiovasc. Pharmacol. Ther. *22*, 3–19. https://doi.org/10.1177/1074248416651722.

Yu, J.K., Franceschi, W., Huang, Q., Pashakhanloo, F., Boyle, P.M., and Trayanova, N.A. (2019). A comprehensive, multiscale framework for evaluation of arrhythmias arising from cell therapy in the whole post-myocardial infarcted heart. Sci. Rep. *9*, 9238. https://doi.org/10.1038/s41598-019-45684-0.

Stem Cell Reports, Volume *16*

Supplemental Information

Pharmacologic therapy for engraftment arrhythmia induced by trans-plantation of human cardiomyocytes

Kenta Nakamura, Lauren E. Neidig, Xiulan Yang, Gerhard J. Weber, Danny El-Nachef, Hiroshi Tsuchida, Sarah Dupras, Faith A. Kalucki, Anu Jayabalu, Akiko Futakuchi-Tsuchida, Daisy S. Nakamura, Silvia Marchianò, Alessandro Bertero, Melissa R. Robinson, Kevin Cain, Dale Whittington, Rong Tian, Hans Reinecke, Lil Pabon, Björn C. Knollmann, Steven Kattman, R. Scott Thies, W. Robb MacLellan, and Charles E. Murry

Supplemental Figures

Figure S1

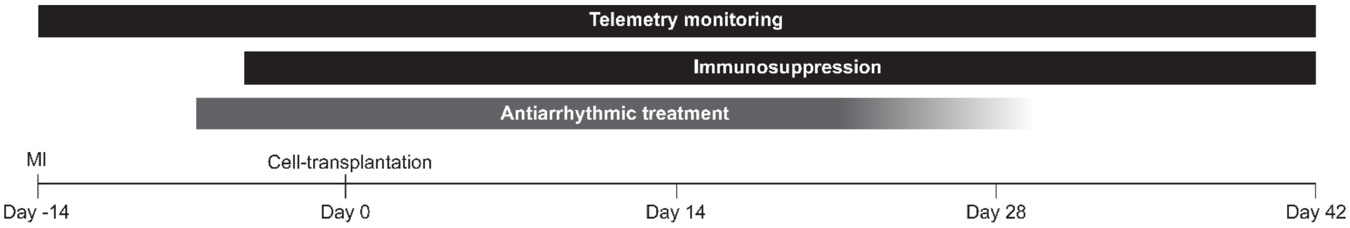

**Immunosuppression**	
Calcineurin inhibitor	Cyclosporine PO (trough goal >300 ng/mL)
Glucocorticoid	Methylprednisolone 1.5 mg/kg PO QD x2 week, 1 mg/kg thereafter
CTLA4 inhibitor	CTLA4-Ig (abatacept) 12.5 mg/kg IV q 2 weeks
Prophylaxis	Cephalexin, sulfamethoxazole/trimethoprim, ganciclovir, probiotics

**Antiarrhythmic treatment**	
Rhythm/rate control	Amiodarone 200–1200 mg BID PO (steady state serum goal 1.5–2.5 µg/mL)
Rate control	Ivabradine 2.5–15 mg PO BID for HR ≥150 bpm (goal <125 bpm)

Study timeline for Phase 2 drug trial of chronic amiodarone and adjunctive ivabradine therapy. Myocardial infarction (MI) was induced by 90-minute balloon occlusion of the mid-left anterior descending artery two weeks prior to human embryonic stem cell-derived cardiomyocyte transplantation (day 0). All subjects received multi-drug immunosuppression. Treated cohort received rate and rhythm control with combined oral amiodarone and adjunctive oral ivabradine.

Figure S2

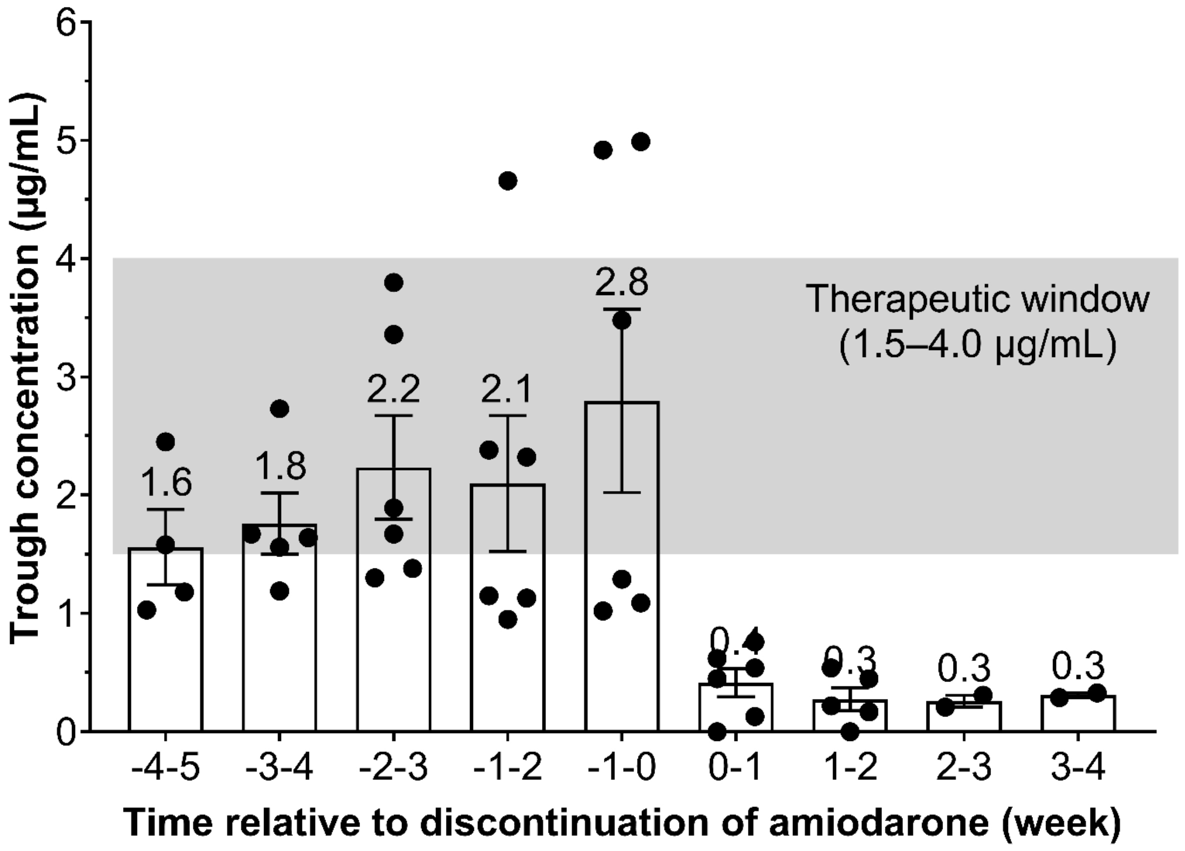

Plasma amiodarone levels in pigs. Amiodarone levels were measured in plasma by a custom liquid chromatography-mass spectrometry assay. Chronic oral amiodarone in six pigs was discontinued after achieving electrical maturation and stabilization of engraftment arrythmia. Serum through concentrations of amiodarone were assayed weekly including 3–4 weeks after discontinuation.

Figure S3

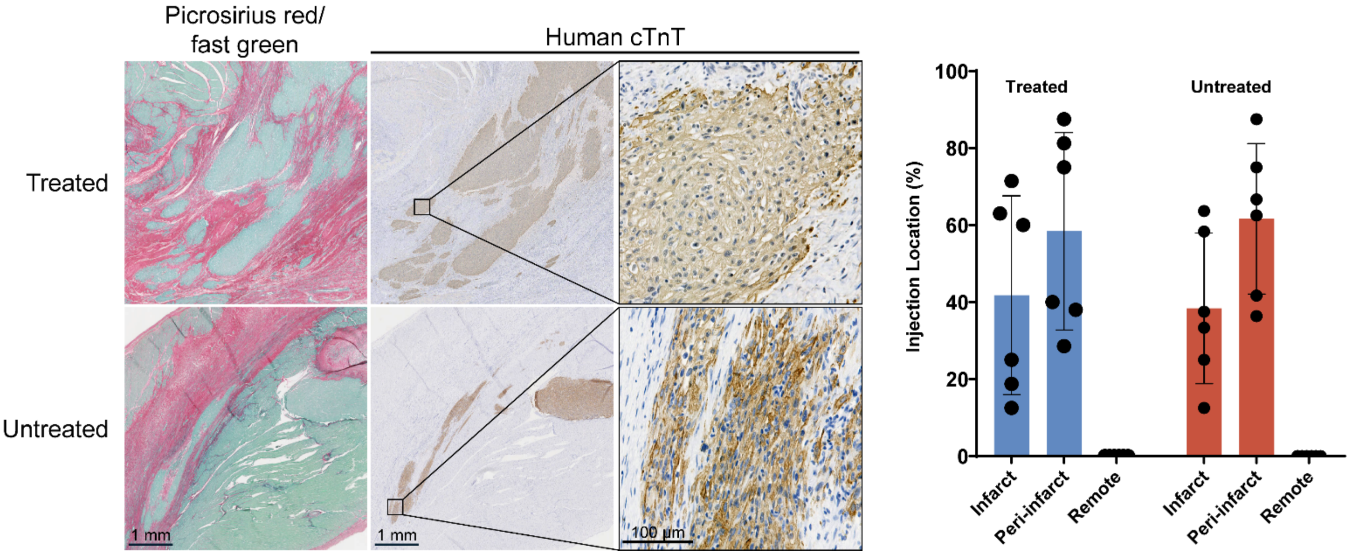

hESC-CM graft histology and location. Left panel: Histological sections stained with picrosirius red to identify collagen (infarct) and fast green to identify viable myocardium. Adjacent sections labeled with human cTnT (brown) identify transplanted hESC-CM graft within unstained porcine myocardium and scar tissue. Sections from both treated and untreated subjects were obtained on post-transplantation day 42. Right panel: Transplanted hESC-CM grafts were located similarly between treated (blue) and untreated (red) cohorts and successfully targeted the infarct and peri-infarct regions of the anterior wall.

Figure S4

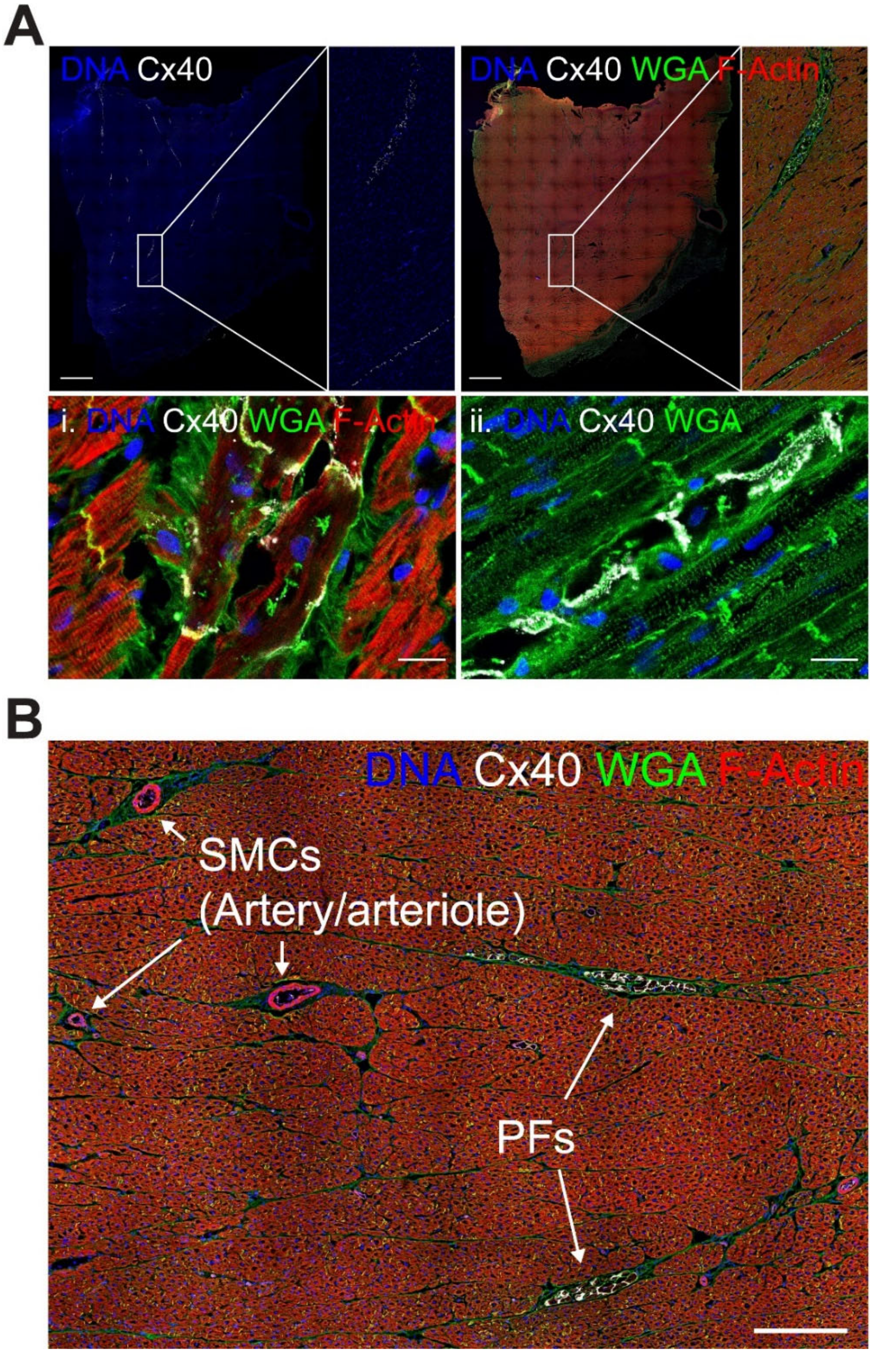

Purkinje fibers are distributed in a mesh-like network throughout the native porcine myocardium and are specifically marked by Connexin 40. Subendocardial and intramyocardial connexin 40 (Cx40)-positive Purkinje fibers (PFs, white) in transverse section of left ventricular free wall, scale bar 2 mm (A). Intramyocardial PFs are shown with higher magnification insets. Further magnified view of white boxed regions show Cx40 localizes to gap junctions of Purkinje cells that display lower sarcomere content (F-Actin, red) (i.) and lack T-Tubules (WGA, green) (ii.) in contrast to surrounding cardiomyocytes, scale bar 20 µm. Cx40 specifically marks myocardial Purkinje fibers (PFs) diffusely distributed in the anterior left ventricular wall cross section, scale bar 2 mm (B).

Supplemental Table 1

**Lidocaine (Ib)**	100 mg IV	Modest HR effect, rare cardioversion
**Flecainide (Ic)**	2 mg/kg PO, 4 mg/kg PO, 6 mg/kg PO, 10 mg/kg PO	No response on HR or EA burden
**Propafenone (Ic)**	1 mg/kg IV, 2 mg/kg IV, 3 mg/kg IV	No response on HR, transient cardioversion*
**Amiodarone (III)**	150 mg IV	Modest HR effect, frequent cardioversion
**Sotalol (III)**	1 mg/kg PO, 2 mg/kg PO, 4 mg/kg PO	No response on HR or EA burden
**Metoprolol (β_1AR)**	5 mg IV, 25 mg PO BID, 50 mg PO BID, 75 mg PO BID	Moderate HR effect (IV only), no response on EA burden
**Ivabradine (I_f)**	2.5 mg PO, 5 mg PO BID, 10 mg PO BID, 15 mg BID; 1 mg/kg IV, 2 mg/kg IV	Robust dose-dependent HR effect (PO only)**, no response on EA burden

* Severe nausea/emesis observed at therapeutic doses, limiting clinical utility

** Severe bradycardia

Abbreviations: β_1AR, β1-adrenergic receptor; BID, twice daily; HR, heart rate; EA, engraftment arrythmia; I_f, funny current; PO, oral; VT, ventricular tachycardia

Supplemental Experimental Procedures

Animal subject care

All protocols were approved and conducted in accordance with the University of Washington (UW) Office of Animal Welfare and the Institutional Animal Care and Use Committee. Animals received ad libitum water and were fed twice a day (Lab Diet-5084 Laboratory Porcine Grower Diet). For surgical procedures, anesthesia was induced with a combination of intramuscular butorphanol, acepromazine and ketamine. Animals were intubated and mechanically ventilated using isoflurane and oxygen to maintain a surgical plane of anesthesia. Vital signs were monitored continuously throughout each procedure. All animals received subcutaneous Buprenorphine SR-Lab (ZooPharm) for post-operative analgesia and were euthanized by intravenous Euthasol (Virbac). All post-mortem examinations were performed by a blinded board-certified veterinary pathologist.

Porcine myocardial infarction model

Percutaneous ischemia/reperfusion injury was induced as previously described in NHP [1] with modification for the porcine model. A 5–8 cm incision was made in the femoral triangle and the femoral artery was exposed by blunt dissection. Prior to obtaining vascular access, heparin was administered to achieve therapeutic anticoagulation (activated coagulation time > 250 sec). A 5-French guidewire/introducer sheath system (Terumo Medical) was placed into the femoral artery and secured. Continuous ECG, invasive arterial blood pressure, pulse-oximetry and capnography were monitored throughout the procedure. Intravenous amiodarone 150 mg and lidocaine 100 mg were administered as single boluses prior to ischemia to minimize the risk of arrhythmia. Under fluoroscopic guidance (OEC 9800 Plus, GE Medical Systems), a 5-French Judkins right 2 or hockey stick guide catheter (Boston Scientific) was advanced into the ascending aorta to selectively engage the ostium of the left main coronary artery. Coronary angiography was performed using hand injections of contrast (Visipaque) and a 0.014” coronary guidewire (Runthrough NS Extra Floppy, Terumo Medical) was placed into the distal left anterior descending coronary artery (LAD). An appropriately sized angioplasty balloon catheter was then positioned into the mid-LAD distal to the first diagonal branch artery and inflated to the minimum pressure required for total obstruction of distal perfusion as confirmed by angiography. Ischemia was confirmed by ST-segment elevation on the ECG. Animals were maintained under anesthesia with ventilatory and hemodynamic support for 90 minutes, after which the balloon was deflated to restore distal perfusion, again confirmed by fluoroscopy and ECG. The animal was observed for reperfusion arrhythmias and externally cardioverted if ventricular fibrillation occurred. Prior to recovery, all animals received implantable telemetry units and central venous catheter placement. Briefly, the external jugular vein in the jugular furrow was exposed and a 5-French central venous catheter (Access Technologies) was inserted and tunneled out to the dorsal prescapular area. The telemetry transmitter (EMKA easyTEL+) was implanted in a subcutaneous pocket using the same incision in the jugular furrow, and subcutaneous leads were tunneled to capture the cardiac apex to base. The overall procedural mortality including the infarct was < 10%.

Phase 1 study design

Five subjects were administered serial trials of antiarrhythmics (Supplemental Table 1) and observed for acute response by continuous ECG monitoring with the following dosing schedule:

**Subject A**	Ivabradine	Sotalol	Flecainide	Metoprolol	Amiodarone
**Subject B**	Sotalol	Metoprolol	Flecainide	Ivabradine	Lidocaine

**Subject C**	Lidocaine	Ivabradine	Amiodarone		
**Subject D**	Propafenone	Amiodarone			
**Subject E**	Propafenone	Amiodarone			

Antiarrhythmics were trialed after at least two continuous days of EA and continued until the subject became clinical unstable. Dose escalation of the same intravenous agents were delivered as a bolus dose over two minutes with a minimum of 10 minutes between doses through an indwelling central venous catheter. Oral agents were administrated by direct observation in a minimum of apples, apple sauce or pumpkin puree with daily feeding and titrated daily for dose escalation. A washout period of at least three days was provided between agents. Amiodarone was administered as the last agent for testing given concern for prolonged half-life and elimination kinetics. Cardioversion was observed with amiodarone and propafenone, typically immediately or within minutes of intravenous dose administration.

Purkinje fiber histology

For thin sections, tissue was cut and trimmed to 1 cm × 1 cm × 3 mm, snap frozen in isopentane, and embedded in OCT (TissueTek). 10 µm sections were immersed in 100% methanol at -20°C for 15 minutes and stained with standard immunofluorescence technique using stains described below. Images were acquired on a Leica SP8 confocal microscope.
For thick sections, 1 cm × 1 cm × 3 mm pieces of tissue containing graft were incubated in 100% methanol at 20°C for 1 hour, rehydrated (80% methanol, 60% methanol, 0% methanol, diluted in PBS, 15-minute incubation at -20°C for each reagent). 150 µm sections were cut on a Leica VT1200s vibratome and stained with standard immunofluorescence technique using stains described below. Stained sections were then cleared using BABB as previously reported (El-Nachef et al., 2018), and imaged on a Leica SP8 confocal microscope with 1 µm z-step increments.

Purkinje fiber staining

Sections were stained the following reagents: Hoechst 33342 (DNA, Thermo Fisher Scientific, #62249), Wheat germ agglutinin-Oregon Green (WGA, Thermo Fisher Scientific, #W6748), Phalloidin-647 (F-Actin, Thermo Fisher Scientific, #A22287), anti-Connexin 40 (Cx40, Alpha Diagnostics, #CXN40A), or anti-slow skeletal troponin I (ss-TnI, Novus, #NBP2-46170) with one of two anti-rabbit secondary antibodies (Alexa Fluor 555/647, Thermo Fisher Scientific, #A-31570/A-31573).

References

1. Liu YW, Chen B, Yang X, Fugate JA, Kalucki FA, Futakuchi-Tsuchida A, Couture L, Vogel KW, Astley CA, Baldessari A, Ogle J, Don CW, Steinberg ZL, Seslar SP, Tuck SA, Tsuchida H, Naumova AV, Dupras SK, Lyu MS, Lee J, Hailey DW, Reinecke H, Pabon L, Fryer BH, MacLellan WR, Thies RS and Murry CE. Human embryonic stem cell-derived cardiomyocytes restore function in infarcted hearts of non-human primates. *Nat Biotechnol*. 2018;36:597-605.
2. Staubli M, Bircher J, Galeazzi RL, Remund H and Studer H. Serum concentrations of amiodarone during long term therapy. Relation to dose, efficacy and toxicity. *Eur J Clin Pharmacol*. 1983;24:485-94.
3. Mostow ND, Rakita L, Vrobel TR, Noon DL and Blumer J. Amiodarone: correlation of serum concentration with suppression of complex ventricular ectopic activity. *Am J Cardiol*. 1984;54:569-74.
4. Chong JJ, Yang X, Don CW, Minami E, Liu YW, Weyers JJ, Mahoney WM, Van Biber B, Cook SM, Palpant NJ, Gantz JA, Fugate JA, Muskheli V, Gough GM, Vogel KW, Astley CA, Hotchkiss CE, Baldessari A, Pabon L, Reinecke H, Gill EA, Nelson V, Kiem HP, Laflamme MA and Murry CE. Human embryonic-stem-cell-derived cardiomyocytes regenerate non-human primate hearts. *Nature*. 2014;510:273-7.
5. Schindelin J, Rueden CT, Hiner MC and Eliceiri KW. The ImageJ ecosystem: An open platform for biomedical image analysis. *Mol Reprod Dev*. 2015;82:518-29.
6. Deroulers C, Ameisen D, Badoual M, Gerin C, Granier A and Lartaud M. Analyzing huge pathology images with open source software. *Diagn Pathol*. 2013;8:92.
7. El-Nachef D, Oyama K, Wu YY, Freeman M, Zhang Y and MacLellan WR. Repressive histone methylation regulates cardiac myocyte cell cycle exit. *J Mol Cell Cardiol*. 2018;121:1-12.
